# Everolimus suppresses glucose transporter 3 membrane trafficking to improve therapeutic efficacy of umbilical cord blood-derived mesenchymal stem cell transplantation in diabetic retinopathy

**DOI:** 10.1038/s41419-026-08673-6

**Published:** 2026-03-28

**Authors:** Hyo Youn Jo, Ji Seung Jung, Hang Hyo Jo, Dae Hyun Kim, Yeon Ju Oh, Jiyi Hwang, Kyung-Mee Park, Hyun Jik Lee

**Affiliations:** 1 Laboratory of Veterinary Physiology, College of Veterinary Medicine and Veterinary Medicine Center, Chungbuk National University, Cheongju, Republic of Korea; 2 Institute for Stem Cell & Regenerative Medicine (ISCRM), Chungbuk National University, Cheongju, Republic of Korea; 3 Laboratory of Veterinary Surgery and Ophthalmology, College of Veterinary Medicine and Veterinary Medicine Center, Chungbuk National University, Cheongju, Republic of Korea

**Keywords:** Mesenchymal stem cells, Actin, Apoptosis, Nutrient signalling

## Abstract

Diabetic retinopathy (DR) is a microvascular and retinal neurologic disorder that occurs in patients with long-term diabetes. Umbilical cord blood-derived mesenchymal stem cell (UCB-MSC) therapy has emerged as a promising treatment because of its regenerative potential; however, its effectiveness is limited under hyperglycemic conditions, which results in the overproduction of mitochondrial reactive oxygen species (mtROS), leading to cellular senescence. In this study, we examined the potential of everolimus, a mammalian target of rapamycin (mTOR) inhibitor, to enhance the efficacy of MSCs in a high glucose environment, which is typical of DR. Increased glucose levels enhanced glucose uptake, primarily through glucose transporter 3 (GLUT3) overexpression, which resulted in the excess generation of mtROS and ultimately induced cell death. Everolimus inhibited intracellular glucose levels and mtROS production, and increased the survival of MSCs under high glucose conditions. Everolimus also inhibited mTORC1, which resulted in reduced actin stabilization and decreased membrane translocation of GLUT3. This effect was associated with the down-regulation of cofilin phosphorylation, a key factor in actin dynamics, which further suppressed high glucose-induced glucose influx and mtROS generation. Furthermore, the results of the streptozotocin (STZ)-induced DR rat model indicated that the groups receiving a subconjunctival injection of *GLUT3* knockdown or everolimus-pretreated UCB-MSCs in STZ rats showed improved retinal function compared with the untreated DR groups. Taken together, the results suggest that everolimus enhances the viability and function of UCB-MSCs under hyperglycemic conditions by modulating glucose homeostasis and reducing oxidative stress. This represents a novel therapeutic strategy for the treatment of DR.

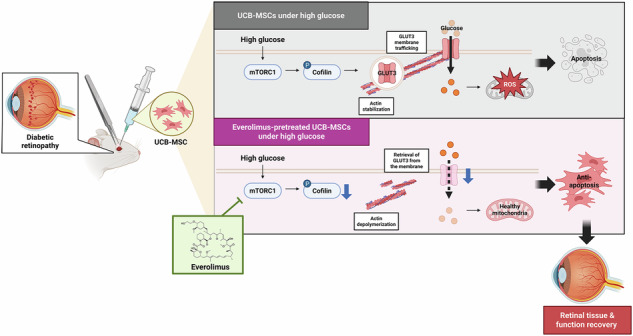

## Introduction

Diabetic retinopathy (DR) is a common microvascular complication that leads to blindness in patients with long-term diabetes [1]. Approximately 20% of individuals who have had diabetes for more than ten years develop diabetic macular edema, which is worsened by a lack of interaction between retinal nerve cells, glial cells, and blood vessels (known as neurovascular units) during early-stage diabetes [2]. The first-line treatment for DR is anti-VEGF therapy, which has disadvantages, such as insufficient prophylactic effect and recurrence of the pathogenesis. Thus, a new therapeutic approach is needed based on a functional and tissue regeneration perspective [3]. Cell therapy with mesenchymal stem cells (MSCs) is considered a promising treatment for restoring vision loss because of its paracrine trophic effects, cell differentiation, and immunomodulatory properties [4]. Nevertheless, the stemness and viability of MSCs are reduced under hyperglycemic conditions, which reduces the success of engraftment compared with normal conditions [5]. Cellular senescence caused by mitochondrial dysfunction and increased mitochondrial reactive oxygen species (mtROS) as a result of high glucose conditions has been identified as the primary cause [6, 7]. Consequently, a mitochondria-based strategy that alleviates mtROS under high glucose conditions is needed to improve the therapeutic efficacy of MSC transplantation into DR patients.

mtROS is a major source of cellular reactive oxygen species (ROS) produced within cells. They occur naturally as a byproduct of oxygen respiration in aerobic organisms and are closely related to glucose metabolism [8]. When excess glucose enters the cell, it is digested in the mitochondria to generate excess NADH and FADH_2_, which accelerates the electron transport chain, raises the risk of electron leakage, and ultimately generates ROS [9]. Moreover, a metabolomic analysis of the Zucker Diabetic Fatty rat model revealed that activated oxidative phosphorylation (OXPHOS) compensates for the loss of adenylate energy charge and NAD^+^ because of disruptions in certain metabolic processes, including glycolysis, but increases ROS production [10]. Therefore, reducing intracellular glucose levels is important in diabetics to prevent the generation of mtROS that results from excessive glucose metabolism. Glucose transporters (GLUTs) are the main pathway for glucose to enter cells. High glucose levels were shown to stimulate the expression of GLUTs in human peritoneal mesothelial cells [11]. When mouse photoreceptor cells were treated with high glucose, an increased Wnt / β-catenin / HIF1α axis upregulates GLUT1 expression and triggers metabolic changes that contribute to oxidative stress, accelerating photoreceptor cell damage [12]. Therefore, the results indicate that maintaining intracellular glucose homeostasis through GLUTs regulation will improve the engraftment of MSCs, although no studies have been reported to date.

Everolimus is a drug that binds to FKBP12 and significantly inhibits mammalian target of rapamycin (mTOR) complex 1 (mTORC1). It has been approved by the FDA as an anti-cancer drug [13]. mTOR is a key signaling molecule in cell survival, proliferation, and glucose metabolism, as it regulates metabolic shifts between glycolysis and OXPHOS in a cell-specific manner [14, 15]. Similarly, several studies reported that everolimus broadly modulates intracellular metabolic switches, including glycolysis and the tricarboxylic acid cycle [16, 17]. Moreover, the mTOR pathway is involved in regulating the expression and translocation of GLUTs to the cell membrane, which is increased under high glucose conditions [18, 19]. In trabecular meshwork cells with severe oxidative stress and mitochondrial damage, mTOR inhibitors promote autophagy to maintain healthy mitochondria and avert cell death [20]. In addition, a previous study demonstrated a mitochondrial anti-oxidative effect of everolimus in senescent T cells, suggesting its potential to inhibit hyperglycemia-induced senescence of transplanted MSCs in DR [21]. However, the detailed mechanism by which everolimus regulates glucose homeostasis and modulates the efficacy of MSCs in DR treatment remains unclear. Therefore, we investigated the therapeutic effect of UCB-MSCs pretreated with everolimus on streptozotocin (STZ)-induced DR rat model. Moreover, we further elucidated the detailed mechanism by which everolimus affects intracellular homeostasis and biological function under high glucose conditions.

## Materials and methods

### Materials

Human UCB-MSCs, CEFOgro™ human MSC growth (CEFOgro™) medium with supplements, penicillin, and streptomycin (#CEFO-UCMSC-kit) were purchased from CEFO (Seoul, Republic of Korea). Phosphate-buffered saline (PBS, #SH30256.01) and Minimal Essential Medium alpha modification (MEM α, #SH30265.01) were obtained from Hyclone (Logan, UT). D-glucose (#G5767), L-glucose (#G5500), antimycin A (#A8674), and cytochalasin D (#C8273) were purchased from Sigma-Aldrich (St. Louis, MO). Everolimus (#1268976) and sirolimus (#1612765) were purchased from the United States Pharmacopeia (Rockville, MD). Bax (#sc-7480), Bcl-2 (#sc-7382), GLUT1 (#sc-377228), Akt (#sc-5298), p-Akt (Ser 473) (#sc-514032), cofilin (#sc-376476), p-cofilin (Ser 3) (#sc-271921), GAPDH (#sc-32233), and β-actin (#sc-47778) antibodies were purchased from Santa Cruz Biotechnology (Dallas, TX). GLUT3 (#NBP2-66872), p-Akt (Thr 308) (#NBP1-69924), and LC3B (#NB100-2220) antibody were purchased from Novus Biologicals (Littleton, CO). Na^+^-K^+^ ATPase α1 (#23565S), mTOR (#2983S), p-mTOR (Ser 2448) (#5536S), p70 S6 Kinase (#2708S), p-p70 S6 Kinase (Thr 389) (#9234S), and cleaved caspase-9 (#95077S) antibodies were purchased from Cell Signaling Technology (Beverly, MA). All PCR primers for *GLUT*, *GLUT3*, *SOD1*, *SOD2*, and *CAT* were synthesized by Cosmogenetech (Seoul, Republic of Korea). Small interfering RNA (siRNA) for *GLUT1* and *GLUT3*, and non-targeting (NT) siRNA were obtained from Bioneer (Daejeon, Republic of Korea).

### Cell culture

Human UCB-MSCs were cultured in CEFOgro^TM^ medium supplemented with 10% fetal bovine serum and 0.5% penicillin and streptomycin. The cells were incubated at 37 °C in a 5% CO_2_ atmosphere. After the cells reached approximately 60–70% confluency, the medium was switched to MEM α medium supplemented with 1% penicillin and streptomycin (Hyclone, Logan, UT, #SV30010) for 24 h. In the time-dependent D-glucose exposure experiments, a fixed-endpoint design was employed to eliminate variables associated with total culture duration. All cells were subjected to serum starvation for a total of 72 h. High glucose (25 mM D-glucose) was added to the treatment groups at designated time points (72, 48, and 24 h) prior to a synchronized harvest. The control group was maintained in serum-free medium for the full 72 h duration without D-glucose supplementation.

### Lactate dehydrogenase (LDH) cytotoxicity assay

The LDH release assay kit (EZ-LDH^TM^; DoGenBio, Seoul, Republic of Korea, #DG-LDH1000) was used for measuring cytotoxicity. UCB-MSCs were seeded into 96-well plates at a density of 1.5 × 10^4^ cells /well. The cell supernatant was centrifuged and mixed with LDH reaction mixture. Using a Synergy^TM^ HTX Multimode Reader (Agilent Technologies, Santa Clara, CA, #S1LFA), the absorbance of the reactants was measured at 450 nm.

### Water-soluble tetrazolium salt (WST-1) cell proliferation assay

The EZ-cytox^TM^ enhanced cell viability assay kit (EZ-Cytox^TM^; DoGenBio, Seoul, Republic of Korea, #EZ-3000) was used to assess cell viability. After removing the cell culture supernatant, the cells adhering to the bottom of the plate were mixed with WST-1 reaction solution and incubated at 37 °C. The absorbance of the reactants was determined at 450 nm.

### Trypan blue exclusion assay

The cells were washed once with PBS and detached with a 0.05% trypsin solution. The cell suspension was mixed with trypan blue (Sigma-Adrich, #T8154) in a 1:1 ratio to stain dead cells. The Countess^TM^ II FL automated cell counter (Thermo Fisher, Waltham, MA, #AMQAF1000) and cell counting chamber slides (Thermo Fisher, #C10283) were used to count the cells.

### Measurements of cellular ROS, mtROS and membrane potential

CM-H2DCFDA (Invitrogen, #C6827) was used to measure cellular ROS. The MitoSOX™ Red mitochondrial superoxide indicator (Thermo Fisher, #M36008) and Tetramethylrhodamine ethyl ester perchlorate (TMRE; Sigma-Aldrich, #871917) were used to measure mtROS and mitochondrial membrane potential, respectively. After washing the cells with FluoroBrite™ (Thermo Fisher, #A1896701), they were incubated for 30 min in a 1 μM CM-H2DCFDA solution, for 5 min in a 5 μM MitoSOX™ solution, or for 15 min in a 200 nM TMRE solution. The stained cells were detected using a Synergy^TM^ HTX Multimode Reader set to 530 and 590 nm for excitation and emission, respectively. For live cell imaging, cells were incubated for 30 min in a mixture containing 1 μM MitoSOX™ and 100 nM MitoTracker™ Green (Thermo Fisher, #M7514). Subsequently, they were exposed to 20 μM Hoechst 33342 (Thermo Fisher, #62249) for 10 min. Fluorescent-stained samples were visualized using a confocal microscope (Leica Microsystems, Wetzlar, Germany). Quantification of mitochondrial superoxide levels was performed using Fiji software (version 2.1.0 / 1.53c; National Institutes of Health, Bethesda, MD) by measuring the mean MitoSOX^TM^ intensity within the mitochondria region.

### Glucose uptake assay

A glucose uptake assay kit (Abcam, Cambridge, UK, #ab136955) was used to quantify intracellular glucose levels based on the manufacturer’s instructions. Briefly, a serum-free medium was applied to cells grown in 96-well plates overnight. After Krebs-Ringer-Phosphate-HEPES buffer supplemented with 2% bovine serum albumin, the samples were treated with 2-deoxyglucose and rinsed with PBS. Extraction buffer was added to lyse the cells, followed by neutralization buffer, and centrifugation. The supernatant was transferred to a fresh tube and diluted 10-fold with assay buffer. The reaction buffer was added to the samples. The extraction-neutralization procedure was carried out to eliminate any leftover nicotinamide adenine dinucleotide phosphate. After adding the reaction buffer to the sample, the absorbance was measured at a wavelength of 412 nm.

### Subcellular fractionation

The EzSubcell^TM^ subcellular fractionation/extraction kit (Atto, Tokyo, Japan, #WSE-7421) was used for membrane fractionation. The extraction mixture, prepared by mixing the extraction buffer and protease inhibitor, was added to the sample and incubated for 10 min at 4 °C. After centrifugation at 700 × *g* for 5 min, the supernatant was considered the cytosol. The remaining pellet was subject to extraction for 30 min and centrifuged for 5 min at 4000 × *g*. The resulting supernatant was considered the cell membrane fraction.

### Western blot analysis

To obtain whole cell lysates, RIPA lysis buffer (Atto, #WSE-7420) was added, and the samples were crushed using an ultrasonic homogenizer. After centrifugation to remove cellular debris, the proteins were quantified using a BCA protein assay kit. The proteins were separated on a 6–10% sodium dodecyl sulfate polyacrylamide gel and transferred to a polyvinylidene fluoride membrane (Hyclone, #GE10600021). The membranes were washed with a Tris-buffered saline solution containing 0.1% Tween 20 (TBST) and blocked with 5% skim milk (LPS solution, Daejeon, Republic of Korea, #SK1500). The membranes were incubated with primary antibody overnight at 4 °C, washed, and then incubated with anti-mouse or anti-rabbit secondary antibody coupled to horseradish peroxidase for 2 h at RT. After washing three times with TBST, the bands were identified using an enhanced chemiluminescence substrate (BIO-RAD, Hercules, CA, #170-5061). Full and uncropped immunoblot images are provided in the Supplementary Materials.

### Real-time quantitative polymerase chain reaction (qPCR)

RNA was extracted using a kit (TaKaRa, Shiga, Japan, #9767 A), and 1 μg of RNA was added to an RT-PCR premix tube (iNtRON biotechnology, Seongnam, Republic of Korea, #25081) and reverse-transcribed into cDNA. The cDNA was amplified using TB Green Premix Ex Taq^TM^ (TaKaRa, Otsu, Japan, #RR820A). The mRNA expression levels of the *GLUT*, *GLUT3*, *SOD1*, *SOD2*, *CAT*, and *ACTB* genes were determined using the CFX^TM^ Connect real-time PCR detection system (BIO-RAD, #1855201). Identification of the PCR products and melting curve analysis was done to assess the specificity, efficiency, and fidelity of the PCR primers. To normalize each gene, we used the *ACTB* gene as a reference. The primer sequences are listed in Supplementary Table S1.

### siRNA transfection

The culture medium was replaced with serum- and antibiotic-free medium for 24 h. For transfection, 25 nM *GLUT1* and *GLUT3* siRNAs were mixed with Turbofect™ transfection reagent (Thermo Fisher, #R0531) in Opti-MEM (Gibco, #51985034) according to the manufacturer’s instructions. The transfection mixture was added to the cells and incubated for 24 h. After incubation in serum-free medium for 72 h, we confirmed the knockdown efficacies of *GLUT1* and *GLUT3* siRNAs by real-time qPCR, showing at least 50% reduction compared to the non-targeting siRNA control (Supplementary Fig. S1). The sequences of the siRNAs are listed in Supplementary Table S2.

### Glucose metabolism microarray

To compare glucose metabolism-related genes expressed in each experimental group, the AccuTarget^TM^ qPCR Screening Kit (Bioneer, #SH-0000-10) was used. A 100 ng aliquot of cDNA was mixed with AccuPower^TM^ 2X GreenStar qPCR Master Mix (Bioneer, #K-6251) and each primer. After a pre-denaturation step at 95 °C for 5 min, a 2-step PCR protocol was carried out as follows: 95 °C for 15 s and 58 °C for 30 s, which was repeated for a total of 55 cycles. Data analysis was performed using R software (version 4.0.2; R Foundation for Statistical Computing, Vienna, Austria) and R Studio (version 2024.04.1; RStudio, PBC, Boston, MA).

### Immunocytochemistry

Cells were grown in confocal dishes (SPL Life Sciences Co., Ltd., Pocheon, Republic of Korea, #10135). After washing three times with FluoroBrite^TM^, the live cells were stained using MemBrite^TM^ Fix (Biotium, Fremont, CA, #30095-T) or MitoTracker™ Green. The samples were fixed in acetone for 5 min and blocked with 1% bovine serum albumin (LPS solution, #9048-46-8) in PBS containing 0.2% Tween-20 (PBST) for 30 min. The samples were then incubated with primary antibody in PBST (1:300 dilution), followed by a fluorochrome-conjugated secondary antibody (1:200). To stain F-actin, the cells were incubated with phalloidin (Cell Signaling Technology, #8878S) for 15 min. To stain the nuclei, the cells were exposed to 4’,6-diamidino-2-phenylindole (DAPI; Sigma-Aldrich, #28718-90-3) or Hoechst 33342. Immunofluorescent-stained samples were visualized using a confocal microscope. To evaluate colocalization between GLUT3 and cellular structures, we measured the ratio of the integrated optical density (IOD) of the yellow area to the total IOD of the red+green+yellow area using Fiji software. For mitochondrial dynamics, mitochondrial morphology (area, length, form factor, and branch junctions) was quantified using the Mitochondrial Analyzer plugin (http://sites.imagej.net/ACMito/). Mitophagy was assessed by quantifying the proportion of mitochondrial area colocalized with LC3 puncta using Fiji.

### Animals

Male Sprague–Dawley (SD) rats (8 weeks old; Nara Biotech, Pyeongtaek, Republic of Korea) were used in two independent in vivo experiments to examine the effects of pretreated UCB-MSCs on retinal function and structure in DR. Rats were housed in a conventional environment under a 12 h light / dark cycle, with food and water available *ad libitum*. For experiments using *GLUT3* knockdown of MSCs (MSC-G), 12 rats were divided into the following four groups: vehicle-injected wild-type rats (group 1, *n* = 3); STZ-induced DR rats (group 2, *n* = 3); and DR rats receiving UCB-MSCs pretreated with either NT siRNA (group 3, *n* = 3) or *GLUT3* siRNA (group 4, *n* = 3). In the experiments using everolimus-pretreated UCB-MSCs (MSC-E), another set of 16 rats was divided into four groups: vehicle-injected wild-type rats (group 1, *n* = 4); STZ-induced DR rats (group 2, *n* = 4); and DR rats receiving UCB-MSCs alone (group 3, *n* = 4) or pretreated with everolimus (group 4, *n* = 4). Group separation was performed at week 9 (MSC-G) and week 10 (MSC-E). Diabetic mellitus (DM) was induced by a single intraperitoneal injection of streptozotocin (STZ, 55 mg/kg body weight) (Sigma–Aldrich, St. Louis, MO, #S0130) dissolved in citrate buffer (pH 4.5) (Sigma–Aldrich, #C2488). Vehicle-injected wild-type rats received an equivalent volume of citrate buffer alone. Blood glucose (BG) levels were measured at intervals post-injection, and rats with BG levels ≥250 mg / dL were considered diabetic. Body weight (BW) and BG were monitored. For the MSC-G experiment, measurements were taken at 0, 3, 6, 9, and 12 weeks post-STZ injection. For the MSC-E experiment, measurements were taken at 0, 3, 10, and 17 weeks. Before group separation, comparisons were made between the vehicle and STZ-injected groups. After separation, the BW and BG levels were compared among the four experimental groups. At the time of separation, the animals were assigned to each group to ensure that no significant differences in BW and BG existed. Rats showing a BW reduction exceeding 30% of their initial BW at any measurement time point were euthanized, and their data were excluded from analysis. Group sizes (3–4 animals per group, 6–8 eyes) were chosen with reference to prior diabetic retinopathy studies, and subgroup allocation was balanced to minimize baseline variability. No blinding was performed, but all procedures followed standardized protocols to reduce potential bias.

### Preparation of UCB-MSCs for transplantation into DR rat models

*GLUT3* siRNA-transfected UCB-MSCs were prepared based on the previously described siRNA transfection protocol. Everolimus was added to the culture media for 24 h. The media was removed, the cells were washed three times with PBS, and detached by incubating with 0.05% trypsin. Cell injections were conducted subconjunctivally in both eyes at 9 and 10 weeks post-STZ injection (MSC-G experiment) and 11 and 12 weeks post-STZ injection (MSC-E experiment). For subconjunctival injections, rats were anesthetized using inhalational isoflurane, ocular surfaces were disinfected with 0.5% povidone-iodine, and topical anesthesia was administered using 0.5% proparacaine hydrochloride (Alcaine; Alcon, Fort Worth, TX). The cells were injected using an insulin syringe (BD Ultra-Fine™ needle 0.3 mL 31 G × 5 / 16″ (8 mm), Becton Dickinson, Franklin Lakes, NJ, #328289). Each injection consisted of 1 × 10⁵ cells suspended in 10 μL PBS per eye. Rats in the vehicle and untreated diabetic retinopathy groups received an equal volume of PBS.

### Electroretinography (ERG) and slit lamp examination

ERG recordings were performed to confirm the development of DR and assess therapeutic efficacy at 8 and 12 weeks (MSC-G experiment) and 10 and 14 weeks (MSC-E experiment) post-STZ injection. Based on a marked reduction in flicker and b-wave amplitudes compared with the vehicle group, animals in the STZ-injected group were further divided into experimental subgroups. At this time, care was taken to ensure that there were no significant differences in ERG parameters among the subgroups. Prior to ERG, cataract progression was evaluated using slit lamp biomicroscopy was performed to evaluate cataract progression and to monitor potential adverse effects, including infection or inflammation related to subconjunctival injection. Cataracts were graded according to commonly used diagnostic criteria as no cataract, incipient, early immature, late immature, and mature. For statistical comparison among groups, these stages were assigned ordinal scores of 0, 1, 2, 2.5, and 3, respectively. Following a 20–30 min dark adaptation period, the rats underwent ERG testing under dim lighting conditions. Pupils were dilated using topical 0.5% tropicamide and 0.5% phenylephrine (Mydrin-P, Santen, Osaka, Japan). ERG responses were recorded using the RETevet™ ERG system (LKC, Gaithersburg, MD) under flash stimulus (8.0 cd × s/m², 2 Hz) and flicker conditions (28.3 Hz). Gold foil corneal electrodes (ERG-Jet™) were applied following topical anesthesia with Alcaine. A platinum needle reference electrode was placed centrally on the forehead, whereas the ground electrode was positioned subcutaneously at the tail base.

### Histological examination

After each experiment, the rats were euthanized, and both eyes from three rats per group in each experiment were enucleated and immediately fixed in BioFix HD (BioGnost, Zagreb, Croatia, #BFHD-X) solution. The eyes were sagittally sectioned through the optic nerve, and the lenses and vitreous were carefully removed. The tissues were processed and embedded in paraffin. Sections were prepared and stained with hematoxylin and eosin. Retinal thickness measurements were taken at two separate points that were equidistant from the optic nerve head in each retinal section. To compensate for positional variation in retinal thickness measurements, total retinal thickness was expressed as a percentage relative to the mean thickness of the vehicle group. The thickness of each retinal layer is presented as a percentage of the total retinal thickness at each measurement point.

### Statistical analysis

For statistical analysis and graphing, SigmaPlot software (version 12; Systat Software, Inc., San Jose, CA) and GraphPad Prism software (version 6.0; GraphPad Software, San Diego, CA) were used, respectively. All quantitative data are presented as the mean ± standard deviation from independent experiments, and samples were randomly assigned to treatment groups. Comparisons between the two experimental groups were performed using a two-tailed Student’s *t*-test. Comparisons between three or more experimental groups were analyzed using a one-way analysis of variance. To account for within-animal correlation of bilateral measurements, a linear mixed-effects model was additionally performed using SPSS software (version 31.0; IBM Corp., Armonk, NY, USA). Pairwise comparisons were adjusted for multiple testing using the Bonferroni method. Cataract scores (0–3) were treated as ordinal data and analyzed using the Kruskal–Wallis test followed by Dunn’s post hoc comparisons. A *p-value* < 0.05 was considered statistically significant.

## Results

### Stimulatory effect of high glucose on glucose influx and mtROS accumulation in UCB-MSCs

**Fig. 1:**
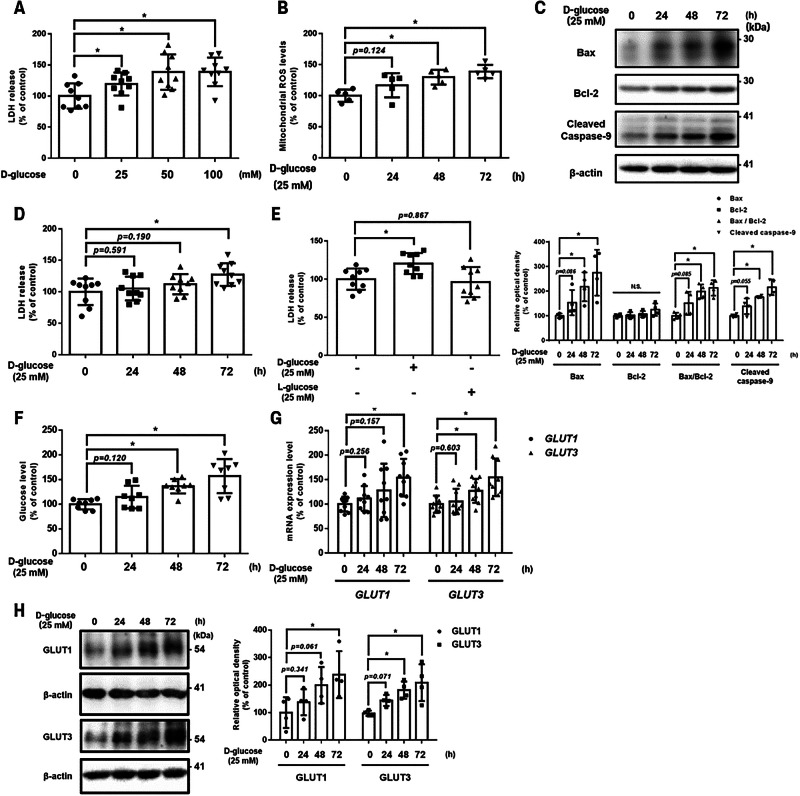
Effects of high glucose on cell death, glucose uptake, and mtROS generation. **A** UCB-MSCs were treated with various concentrations (0–100 mM) of D-glucose for 72 h. Cytotoxicity in the UCB-MSC-conditioned medium was assessed using an LDH release detection kit (*n* = 9). **B–D** UCB-MSCs were treated with D-glucose (25 mM) for 0–72 h. **B** The mtROS generated by the UCB-MSCs was detected using MitoSOX (*n* = 5). **C** Bax, Bcl-2, and cleaved caspase-9 protein expression were determined by western blot analysis (*n* = 4). **D** LDH release was measured to determine cytotoxicity in UCB-MSCs (*n* = 9**). E** UCB-MSCs were treated with 25 mM of D-glucose or 25 mM of L-glucose for 72 h (*n* = 9). **F–H** UCB-MSCs were treated with D-glucose (25 mM) for various times (0–72 h). **F** Glucose transport in UCB-MSCs was measured by the glucose uptake assay (*n* = 8). **G** The expression of *GLUT1* and *GLUT3* mRNA was measured by real-time quantitative PCR (*n* = 9). **H** The expression of GLUT1 and GLUT3 protein was determined by western blot analysis (*n* = 4). All quantitative data are presented as the mean ± standard deviation from independent experiments. *p < 0.05.

The following experiments were conducted to identify the concentration and exposure time of high glucose (i.e., D-glucose) that induced marked changes in UCB-MSCs. LDH release, a cell death marker, was increased at 25–100 mM D-glucose compared with the control (Fig. 1A). Exposure to 25 mM D-glucose for 48–72 h significantly increased intracellular mtROS levels in a time-dependent manner (Fig. 1B). High glucose also induced apoptosis as evidenced by increased Bax and cleaved caspase-9 from 48 h onward (Fig. 1C). Notably, Bcl-2, an anti-apoptotic factor, remained unchanged following exposure to high glucose, which resulted in an increased Bax/Bcl-2 ratio (Fig. 1C). The trypan blue exclusion assay showed a decrease in the high glucose-treated population over 48 h compared with the untreated group, although the LDH release assay with the same settings showed a significant increase at 72 h (Supplementary Fig. S2 and Fig. 1D). The cytotoxic effect of D-glucose is osmolality-independent, as 25 mM of L-glucose, used to create hyperosmolarity artificially, did not change LDH release compared with the control group (Fig. 1E).

Considering that intracellular glucose levels are a source of mtROS generation, we examined changes in glucose influx into UCB-MSCs following time-dependent exposure to high glucose (Fig. 1F). Intracellular glucose levels were increased in the group exposed to high glucose for more than 48 h compared with the control group (Fig. 1F). The glucose transporters that primarily regulate its influx into MSCs are GLUT1 and GLUT3 [22, 23]. The mRNA and protein expression levels for both transporters were increased with longer D-glucose exposure time (Figs. 1G, H); however, GLUT3 showed a significantly higher expression level at 48 h, preceding GLUT1, which responded at 72 h compared with the controls (Figs. 1G, H). Accordingly, high glucose conditions stimulated intracellular glucose influx, accompanied by the up-regulation of both GLUT1 and GLUT3 in UCB-MSCs.

### Protective effects of GLUT3 silencing on UCB-MSCs under high glucose conditions and in DR

**Fig. 2:**
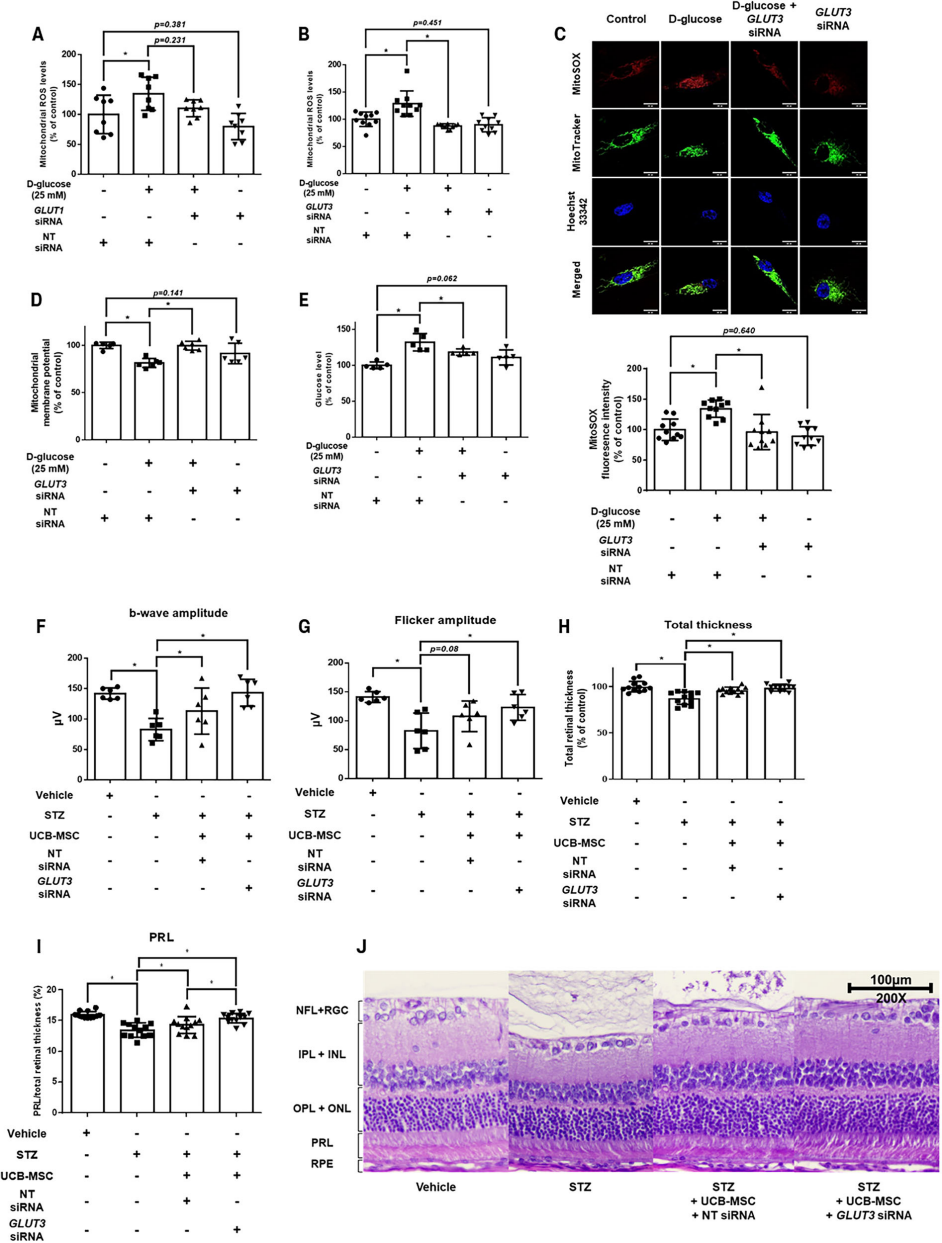
Role of GLUT3 in mtROS production and cell survival under high glucose. **A** UCB-MSCs were transfected with *GLUT1* siRNA or NT siRNA for 24 h and exposed to 25 mM of D-glucose for 72 h. Mitochondrial ROS levels were assessed by MitoSOX staining (*n* = 8). **B–E** UCB-MSCs were transfected with *GLUT3* siRNA or NT siRNA for 24 h, followed by exposure to 25 mM of D-glucose for 72 h. **B** Mitochondrial ROS levels were assessed by MitoSOX staining (*n* = 10). **C** Mitochondrial superoxide generation was visualized by live-cell staining. UCB-MSCs were stained with MitoSOX (red), MitoTracker (green), and Hoechst 33342 (blue) (*n* = 10). Magnification × 1000. Scale bars are 25 μm. **D** Mitochondrial membrane potential was assessed by TMRE staining (*n* = 6). **E** Glucose transport in UCB-MSCs was measured by the glucose uptake assay (*n* = 5). **F**,**G** Electroretinography was done to assess therapeutic efficacy at 12 weeks post-STZ injection. It was measured in both eyes of 3 rats in each group. **F** b-wave amplitudes were compared between the groups (*n* = 6). **E** Flicker amplitudes were compared between the groups (*n* = 6). **H–J** Retinal morphology was examined microscopically at 2 locations in both eyes of 3 rats in each group. **H** Total retinal thickness was measured for each experimental group (*n* = 12). **I** The photoreceptor layer was measured for each experimental group (*n* = 12). **J** Representative images of retinal layers in each group. Magnification ×200. Scale bars are 100 μm. All quantitative data are presented as the mean ± standard deviation from independent experiments. *p < 0.05.

To determine the contribution of each GLUT to mtROS generation, we knocked down the expression of *GLUT1* and *GLUT3* in UCB-MSCs under high glucose conditions by siRNA transfection. High glucose-induced mtROS overproduction in UCB-MSCs was independent of GLUT1 expression (Fig. 2A); however, *GLUT3*-silenced UCB-MSCs mitigated the increased mtROS overproduction under high glucose conditions (Figs. 2B, C). Furthermore, they normalized mitochondrial membrane potential and restricted glucose uptake (Figs. 2D, E). *GLUT3* silencing also suppressed high glucose-induced cellular ROS production and attenuated the upregulation of antioxidant enzyme gene (*SOD1*, *SOD2*, and *CAT*) expression (Supplementary Figs. S3A,B).

To evaluate the therapeutic effects of *GLUT3* knockdown in UCB-MSCs (MSC-G) in DR, systemic parameters, retinal function, and histological changes were assessed throughout the experiment. At 3 weeks post-STZ injection, the treated groups exhibited significantly reduced BW and increased BG levels compared with the vehicle-injected wild-type group, confirming the induction of diabetes (Supplementary Figs. S4A and S4B). These differences were significant between the vehicle group and the diabetic groups (rats receiving vehicle, UCB-MSCs transfected with NT siRNA, or MSC-G) after separation into groups at week 8, when retinal dysfunction was confirmed (Supplementary Figs. S4A–E). In short, UCB-MSCs treatment was initiated through local injection, which did not affect systemic parameters, such as BW and BG levels. ERG measurements at 8 weeks post-STZ injection confirmed retinal dysfunction in the untreated DR rats, with a marked reduction in b-wave and flicker amplitudes relative to the vehicle group (Supplementary Figs. S4C–E). Subconjunctival injections of UCB-MSCs were administered at weeks 9 and 10. At 12 weeks, the *GLUT3* siRNA-transfected UCB-MSC group exhibited significant recovery in b-wave and flicker amplitude compared with the untreated DR group (Figs. 2F, G, and Supplementary Fig. S4F). Importantly, although all diabetic groups developed cataracts by the time of ERG recordings, the severity was comparable among groups, indicating that cataracts were unlikely to confound the functional outcomes (Supplementary Figs. S4G–I). Histological examination confirmed retinal thinning in the untreated DR group compared with that in the vehicle group, with significant restoration of total retinal thickness in both NT and *GLUT3* siRNA-transfected UCB-MSC groups (Figs. 2H, J). Analysis of the ratio of each retinal layer to total retinal thickness revealed that the photoreceptor layer (PRL) was significantly reduced in the untreated DR group (Fig. 2I). In contrast, it was considerably restored in the UCB-MSC group, regardless of whether *GLUT3* was knocked down, and the MSC-G group showed greater improvement compared with the NT siRNA-pretreated UCB-MSC group (Fig. 2I). The nerve fiber layer and retinal ganglion cell layer (NFL + RGC) were significantly decreased in the DR group compared with the vehicle group; however, no treatment-related improvement was observed (Supplementary Fig. S4J). No significant differences were observed among the groups in the thickness of the inner plexiform layer and inner nuclear layer (IPL + INL) or outer plexiform layer and outer nuclear layer (OPL + ONL) (Supplementary Figs. S4K,L). Collectively, these findings demonstrate that silencing *GLUT3* in UCB-MSCs protects them from high glucose-induced oxidative stress and enhances their therapeutic efficacy upon transplantation in diabetic retinopathy.

### Suppressive effect of everolimus on high glucose-induced glucose influx, mtROS accumulation and apoptosis of UCB-MSCs

**Fig. 3:**
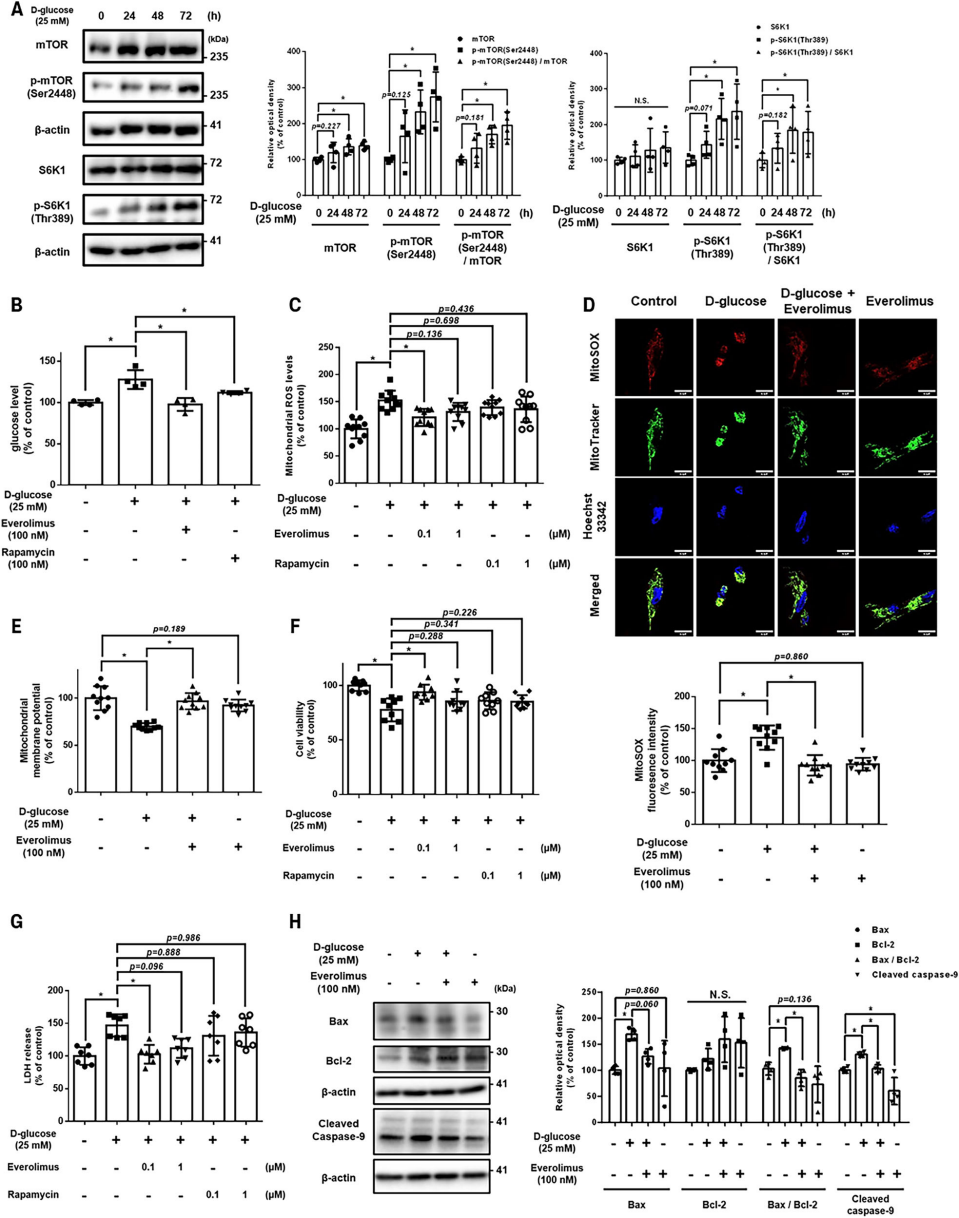
Effect of everolimus on high glucose-induced cell death compared with rapamycin. **A** UCB-MSCs were treated with D-glucose (25 mM) for various times (0–72 h). Protein expression levels of mTOR, p-mTOR (Ser 2448), S6K1, and p-S6K1 (Thr 389) were determined by western blot analysis (*n* = 4). **B** UCB-MSCs were pretreated with 100 nM everolimus or rapamycin for 30 min, followed by D-glucose (25 mM) treatment for 72 h. Intracellular glucose influx in UCB-MSCs was measured by a glucose uptake assay (*n* = 4). **C** UCB-MSCs were pretreated with various concentrations (0.1–1 μM) of everolimus or rapamycin for 30 min followed by D-glucose (25 mM) treatment for 72 h. mtROS levels were assessed by MitoSOX staining (*n* = 10). **D, E** UCB-MSCs were pretreated with everolimus (100 nM) for 30 min followed by D-glucose (25 mM) treatment for 72 h. **D** Mitochondrial superoxide generation was visualized by live-cell staining. UCB-MSCs were stained with MitoSOX (red), MitoTracker (green), and Hoechst 33342 (blue) (*n* = 10). Magnification × 1000. Scale bars are 25 μm. **E** Mitochondrial membrane potential was assessed by TMRE staining (*n* = 10). **F**,**G** UCB-MSCs were pretreated with various concentrations (0.1–1 μM) of everolimus or rapamycin for 30 min followed by D-glucose (25 mM) treatment for 72 h. **F** Cell viability was measured by the trypan blue exclusion assay (*n* = 9). **G** UCB-MSCs were pretreated with various concentrations (0.1–1 μM) of everolimus or rapamycin for 30 min followed by D-glucose (25 mM) treatment for 72 h. Cytotoxicity in UCB-MSC-conditioned medium was measured using an LDH release detection kit (*n* = 7). **H** UCB-MSCs were pretreated with everolimus (100 nM) for 30 min followed by D-glucose (25 mM) treatment for 72 h. Protein expression levels of Bax, Bcl-2, and cleaved caspase-9 were determined by western blot analysis, (*n* = 4). All quantitative data are presented as the mean ± standard deviation from independent experiments. N.S., no significance, *p < 0.05.

UCB-MSCs were treated with high concentrations of D-glucose in a time-dependent manner to determine the role of mTOR signaling in UCB-MSCs under high glucose conditions (Fig. 3A). Based on the ratio of active p-mTOR (Ser 2448) to inactive mTOR, the downstream signaling p-S6K1 (Thr 389)/S6K1 ratio also exhibited a significant increase at 48 and 72 h of high glucose treatment compared with the untreated group (Fig. 3A). These results indicate that a high glucose environment stimulates mTORC1 signaling. Rapamycin binds to FKBP12, similar to everolimus, and was the first FDA-approved mTOR inhibitor for cancer [13]. To identify the most effective drug for controlling intracellular glucose and mtROS production under high glucose conditions, we compared the effects of everolimus and the conventional mTORC1 inhibitor rapamycin in high glucose-treated UCB-MSCs. Everolimus and rapamycin significantly reduced the high glucose-induced intracellular glucose levels (Fig. 3B); however, there was a statistically significant decrease in mtROS production in the group pretreated with 100 nM everolimus, but not rapamycin (Figs. 3C, D). At the same concentration, everolimus reversed the high glucose-induced loss of mitochondrial membrane potential and alleviated cellular ROS overproduction as well as antioxidant enzyme (*SOD1*, *SOD2*, and *CAT*) upregulation (Fig. 3E and Supplementary Figs. S5A,B). High glucose induced excessive mitochondrial fission and mitophagy in UCB-MSCs, as reflected by fragmented mitochondrial morphology and increased colocalization of LC3B with mitochondria. These abnormalities under high glucose conditions were alleviated by everolimus pretreatment, which restored mitochondrial network integrity and reduced mitophagy, thereby supporting mitochondrial quality control (Supplementary Fig. S6). Similarly, in contrast to rapamycin, pretreatment with 100 nM everolimus effectively inhibited high glucose-induced cell death in UCB-MSCs (Figs. 3F, G). The same dose of everolimus also showed anti-apoptotic effects against high glucose (Fig. 3H). These results suggest that low concentrations of everolimus are more effective than rapamycin in attenuating cell death by suppressing glucose levels and mtROS production in UCB-MSCs under high glucose conditions.

### Therapeutic effect of everolimus-pretreated UCB-MSC transplantation in DR rat models

**Fig. 4:**
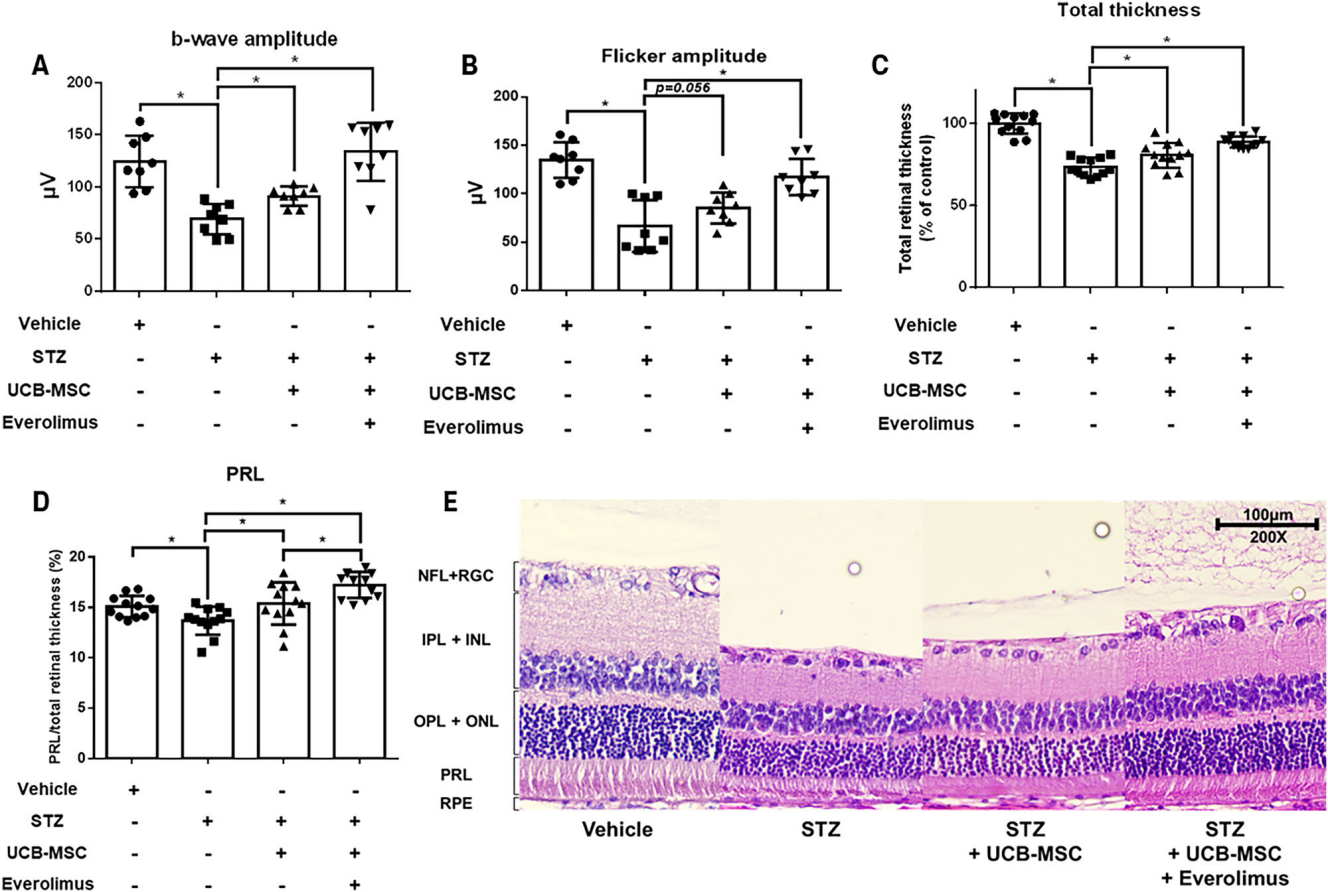
Effect of UCB-MSC transplantation with everolimus treatment in a diabetic retinopathy model. **A, B** ERG was performed to assess therapeutic efficacy at 14 weeks post-STZ injection. It was measured in both eyes of 4 rats in each group. **A** b-wave amplitudes were compared between the groups (*n* = 8). **B** Flicker amplitudes were compared between the groups (*n* = 8). **C–E** Retinal morphology was examined microscopically at 2 locations in both eyes of 3 rats in each group. **C** Total retinal thickness was measured in each group (*n* = 12). **D** The photoreceptor layer was measured for each group (*n* = 12). **E** Representative images of retinal layers in each group. Magnification ×200. Scale bars are 100 μm. All quantitative data are presented as the mean ± standard deviation from independent experiments. *p < 0.05.

A separate in vivo experiment was performed to evaluate the effect of everolimus-pretreated UCB-MSCs (MSC-E) in DR. STZ-induced diabetic rats exhibited significantly reduced BW and elevated BG levels compared with the vehicle-injected wild-type group from the early stages of diabetes induction (Supplementary Figs. S7A,B). This difference remained consistent after the groups were separated (Supplementary Figs. S7A,B). Local administration of UCB-MSCs with or without everolimus did not affect BW and BG. ERG measurements at 10 weeks post-STZ injection confirmed retinal dysfunction in the untreated DR rats, with a marked reduction in b-wave and flicker amplitudes relative to the vehicle group (Supplementary Figs. S7C–E). UCB-MSCs with or without everolimus were administered at 11 and 12 weeks, and ERG was reassessed at 14 weeks post-STZ injection. The group treated with UCB-MSCs alone showed only a modest average increase in flicker amplitude, but a significant improvement in b-wave amplitude (Figs. 4A,B, and Supplementary Fig. S7F). In contrast, the MSC-E group exhibited significant recovery in both parameters compared with the DR group (Figs. 4A,B). By the time of ERG measurements, cataract formation was evident in diabetic groups, but no intergroup differences were observed in severity, suggesting minimal impact on ERG outcomes (Supplementary Figs. S7G and S7H). Histological analysis revealed that total retinal and PRL thickness were significantly reduced in the untreated DR group, but restored in both groups treated with UCB-MSCs (Fig. 4C–E). A greater effect was evident in everolimus-pretreated UCB-MSCs compared with vehicle-pretreated UCB-MSCs (Fig. 4C–E). No treatment-related differences were observed in the NFL + RGC, IPL + INL, or OPL + ONL layers among the groups (Supplementary Figs. S7I–S7K). Taken together, these findings demonstrate that transplantation of everolimus-pretreated UCB-MSCs significantly enhances retinal function recovery and tissue regeneration in DR.

### Regulatory effect of everolimus on cofilin-mediated membrane trafficking of GLUT3 in UCB-MSCs under high glucose conditions

**Fig. 5:**
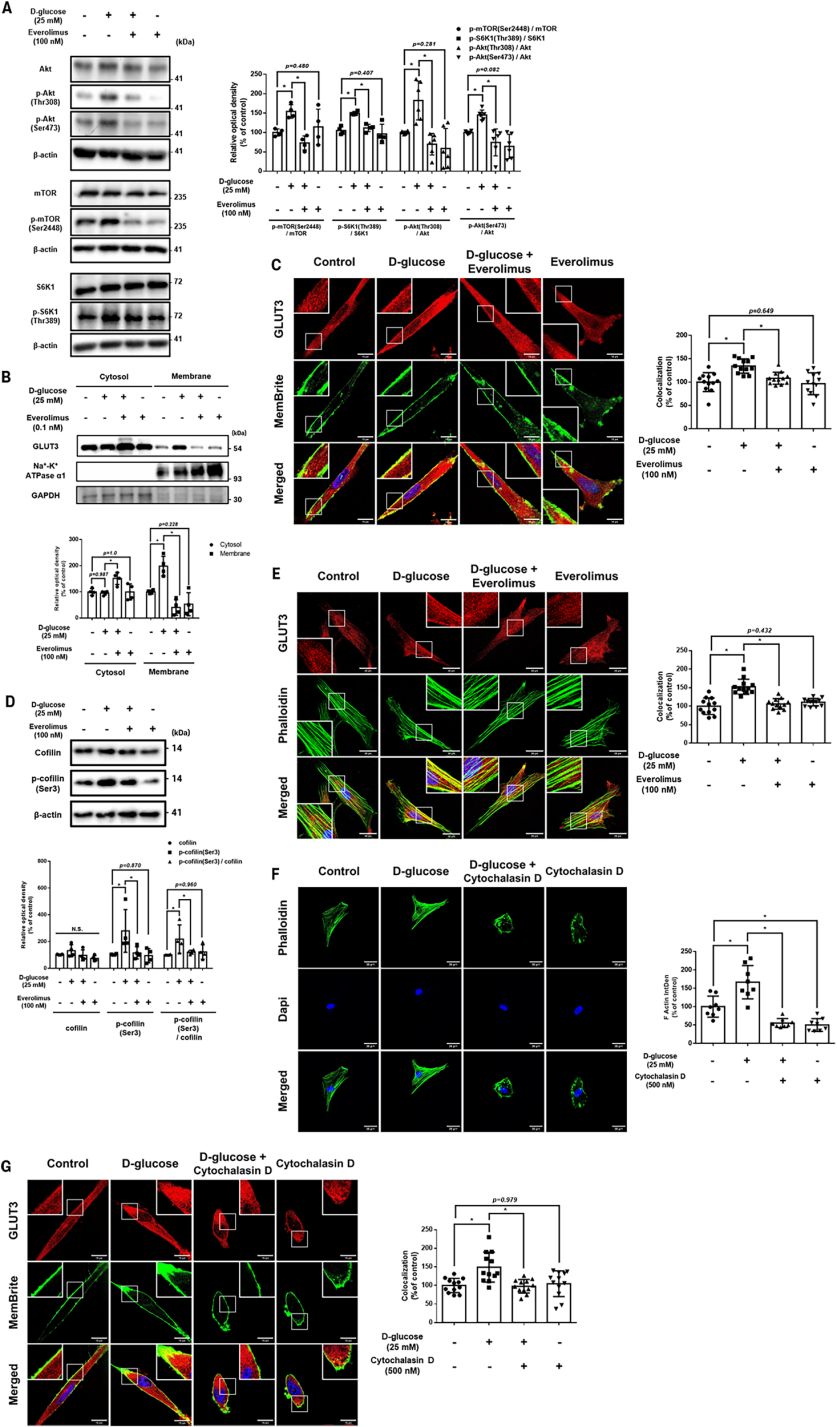
Everolimus-inhibited mTOR signaling suppresses actin-mediated membrane trafficking of GLUT3 under high glucose conditions. **A–E** UCB-MSCs were pretreated with everolimus (100 nM) for 30 min followed by D-glucose (25 mM) treatment for 72 h. **A** The expression ratios of p-mTOR (Ser 2448)/mTOR and p-S6K1 (Thr 389)/S6K1 were confirmed by western blot analysis (*n* = 4). The expression ratios of p-Akt (Thr 308)/Akt and p-Akt (Ser 473)/Akt was also analyzed (*n* = 6). **B** The intracellular fractions of GLUT3 in membranes and cytoplasm were analyzed by western blot analysis (*n* = 4). **C** Membrane trafficking of GLUT3 was visualized by immunocytochemistry. UCB-MSCs were stained with a GLUT3-specific antibody (red), MemBrite (green), and DAPI (blue) (*n* = 12). Magnification × 1500. Scale bars are 15 μm. **D** The expression of cofilin and p-cofilin (Ser 3) was confirmed by western blot analysis (*n* = 4). **E** The physical association of GLUT3 and actin was visualized by immunocytochemistry. UCB-MSCs were stained with GLUT3-specific antibody (red), phalloidin (green), and DAPI (blue) (*n* = 12). Magnification × 1000. Scale bars are 25 μm. **F**, **G** UCB-MSCs were pretreated with cytochalasin D (500 nM) for 30 min followed by D-glucose (25 mM) treatment for 72 h. **F** Actin polymerization was visualized by immunocytochemistry. MSCs were stained with phalloidin (green) and DAPI (blue) (*n* = 8). Magnification × 630. Scale bars are 30 μm. **G** Membrane trafficking of GLUT3 was visualized by immunocytochemistry. UCB-MSCs were stained with a GLUT3-specific antibody (red), MemBrite (green), and DAPI (blue) (*n* = 12). Magnification × 1500. Scale bars are 15 μm. All quantitative data are presented as the mean ± standard deviation from independent experiments. N.S., no significance, *p < 0.05.

The expression of p-Akt (Thr 308) and p-S6K1 (Thr 389), associated with mTORC1 signaling, was increased in high glucose-exposed UCB-MSCs, but was decreased in the everolimus-pretreated group (Fig. 5A). Everolimus also suppressed high glucose-induced p-Akt (Ser 473) which reflect its mTORC2 inhibitory effect (Fig. 5A). To determine whether everolimus modulates glucose metabolism-related enzymes in UCB-MSCs under high glucose conditions, we targeted 54 genes by microarray analysis to determine changes in the presence or absence of everolimus (Supplementary Fig. S8). Examples of genes with statistically significant differences (*p* < *0.05*) that were increased in the everolimus pretreatment group compared with the simple high glucose treatment group included *AGL*, *PGM1*, *PDK1*, and *SUCLA2*. Conversely, genes inactivated by everolimus pretreatment included *MDH1*, *PKLR*, and *PRPS1L1* (Supplementary Fig. S8). The microarray data revealed that everolimus failed to induce overall changes in enzymes involved in glucose metabolism in high glucose-exposed UCB-MSCs. This suggests that the glucose homeostatic effect of everolimus is mediated through the modulation of glucose influx through the transporter, rather than the expression of metabolic enzymes. Therefore, we determined whether everolimus pretreatment affects the regulation of GLUT expression in UCB-MSCs in a high glucose environment (Supplementary Figs. S9A–D). For GLUT1, the high glucose-induced increase in mRNA and protein expression was reversed by everolimus pretreatment (Supplementary Figs. S9A,B). However, as previously shown in Fig. 2, GLUT1 did not contribute to mtROS production in UCB-MSCs. Everolimus pretreatment did not affect GLUT3 expression, but statistically inhibited membrane GLUT3 expression induced by high glucose (Supplementary Figs. S9C,D, and Fig. 5B). This tendency was also reflected in the in immunocytochemical data, where the yellow signal—an interaction between GLUT3-positive and MemBrite-positive fluorescence—increased under high glucose but decreased in the everolimus pretreatment group (Fig. 5C).

The effect of everolimus on actin-mediated GLUT3 membrane translocation was examined in UCB-MSCs under high glucose conditions. The inhibitory phosphorylated form of cofilin (Ser 3), an actin depolymerizing factor, was increased in UCB-MSCs exposed to high glucose and decreased following everolimus pretreatment (Fig. 5D). Consistently, treatment with the mTOR activator MHY1485 also increased the p-cofilin/cofilin ratio, which was reduced by everolimus pretreatment (Supplementary Fig. S10A). In addition, the retrieval of high glucose-induced membrane GLUT3 expression observed in the everolimus-pretreated group was prevented by cotreatment with the actin stabilizer jasplakinolide, resulting in sustained membrane localization of GLUT3 (Supplementary Fig. S10B). These findings further support the mTOR/cofilin/actin remodeling mechanism, indicating that inhibition of mTOR signaling leads to reduced actin stabilization. High glucose increased the overlap between GLUT3-positive fluorescence and phalloidin-stained actin filaments, but this indicator decreased in the everolimus-pretreated group (Fig. 5E). To confirm that actin drives GLUT3 trafficking in UCB-MSCs, cytochalasin D was used to degrade polymerized actin filaments (Figs. 5F,G). Because cytochalasin D causes an imbalance in cytoskeleton dynamics, resulting in cell death, we confirmed that 500 nM cytochalasin D caused cell deformation without killing (Fig. 5F). The colocalization of GLUT3-labeled fluorophores with the cell membrane was increased by high glucose, but decreased by cytochalasin D pretreatment (Fig. 5G). Taken together, when UCB-MSCs are exposed to a high glucose environment, mTOR signaling is activated to stabilize actin structure, which in turn, increases the membrane translocation of GLUT3. However, everolimus inhibits mTOR, which results in the accelerated depolymerization of actin and retrieval of membrane expression of GLUT3.

## Discussion

**Fig. 6:**
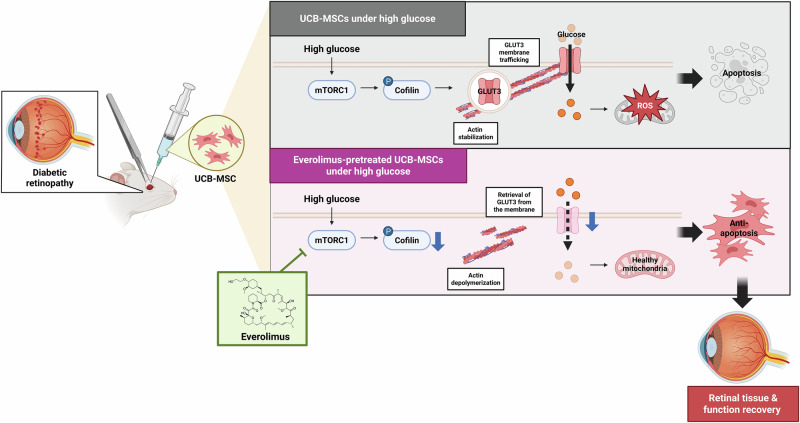
The schematic diagram shows the action mechanism of everolimus pretreatment, which regulates GLUT3 membrane trafficking and mitochondrial anti-oxidation in UCB-MSCs under high glucose conditions. Two types of UCB-MSCs were transplanted subconjunctivally into a rat model of STZ-induced diabetic retinopathy. When UCB-MSCs alone were injected, activated mTORC1 signaling in high glucose conditions increased cofilin phosphorylation, which in turn elongated actin filaments and upregulated the membrane trafficking of GLUT3. The increased membrane expression of GLUT3 allows more glucose to enter the cells, leading to mtROS overproduction. UCB-MSCs pretreated with everolimus inhibited mTORC1 signaling in a high glucose environment, resulting in actin depolymerization via cofilin. Reduced membrane trafficking of GLUT3 results in a reduced intracellular glucose influx, which alleviates mtROS production. In other words, the anti-apoptotic effect of everolimus accelerated retinal tissue and function recovery by UCB-MSCs.

The present study highlights that modulation of GLUT3 trafficking by everolimus, a mTOR inhibitor, reverses the decreased survival and function of transplanted UCB-MSCs in a diabetic retinopathy model (Fig. 6). Under high glucose conditions, excessive mitochondrial respiration and enhanced reduced equivalent influx into the electron transport chain caused electron leakage, which in turn, was associated with mtROS production [9, 24]. Therefore, inhibiting the influx of glucose, the aforementioned metabolic fuel (i.e., modulating GLUTs), represents a strategy to ameliorate high glucose-induced mitochondrial overload and mtROS overproduction [25, 26]. We found that the expression of GLUT1 and GLUT3, the predominant GLUTs in UCB-MSCs, is increased under high glucose conditions; however, the effect of glucose influx via GLUT3 on mtROS production is greater compared with GLUT1. In glomerular epithelial cells, high glucose-induced GLUT1 expression is increased compared with GLUT3, which contributes to impaired energy metabolism, cell hypertrophy, extracellular matrix accumulation, and ROS overproduction [27, 28]. In contrast, the expression of GLUT3 was significantly increased in the lens of diabetic rats, and this increase was observed only in the areas damaged by hyperglycemia [29]. Combined with our results, this suggests that the cells have different profiles and dependencies on GLUT expression depending on the experimental conditions. Specifically, we employed 25 mM D-glucose to mimic the hyperglycemic milieu in vitro [30]. Although this concentration is considered supraphysiological, it has been widely used to induce cellular senescence and reduce proliferation, migration, and osteogenic differentiation potential in mesenchymal stem cells—without causing osmotic effects [30].

mTOR signaling has been implicated as an upstream regulator of GLUTs and mtROS in diabetes-related contexts [18, 31, 32]. Increased nephroblastoma overexpression or cellular communication network factor 3 in blood and tissues and intra-nuclear translocation of METTL3 in gestational DM results in increased GLUT expression through mTOR signaling [18, 31]. Furthermore, in a podocyte of a diabetic kidney disease model, an increase in dihydroxyacetone phosphate resulted in increased ROS production through upregulation of mTORC1, leading to pyroptosis [32]. Although there are reports that mTOR signaling is activated in MSCs exposed to high glucose, the signaling mechanisms that regulate increased GLUTs expression and mtROS production remain unclear [33]. Here, we are the first group to demonstrate that the inhibition of mTOR signaling suppresses GLUT3-induced glucose influx and mtROS accumulation. This indicates that modulating mTOR signaling is important for enhancing the function of UCB-MSCs in the treatment of diabetic complications, including DR.

The present study proposes everolimus as a bio-active drug that inhibits mtROS overproduction and restores the survival and function of UCB-MSCs hindered by high glucose. In rat cardiomyocytes, everolimus enhances autophagy/mitophagy and limits doxorubicin-induced mtROS, thereby regulating mitochondrial homeostasis [34]. In colon cancer and gastric adenocarcinoma cells, however, thioredoxin reductase inhibitors combined with everolimus promote ROS overproduction, thereby activating p-JNK signaling and ER stress to induce cancer cell death [35]. The opposite effects of mTOR inhibitors on oxidative stress was also observed with rapamycin [36]. These results suggest that everolimus exhibits distinct physiological effects depending on the cell-specific physiological characteristics and cellular microenvironment.

mTOR inhibitors have been approved as anti-cancer and immunosuppressive agents. Rapamycin was the first of these drugs that was shown to bind to FBK12 and dissociate Raptor from the mTORC1 complex, thereby inhibiting mTOR signaling [37]. Structurally, everolimus differs from rapamycin by a 2-hydroxyethyl substitution at C-40, which enhances mTORC2 inhibition (Rictor/Sin1 dissociation) and reduces Akt/ERK phosphorylation more than rapamycin [37]. Our results demonstrate that everolimus is more effective in reversing high glucose-induced mtROS production and cell death compared with rapamycin, which leads to improved retinal function and histo-protective effects in vivo. One study has compared the efficacy of rapamycin and everolimus-eluting stents in diabetic patients with cardiovascular disease, with everolimus being associated with fewer vascular complications and improved overall survival [38]. To achieve such robust therapeutic outcomes in our in vitro model, we utilized a concentration of 100 nM everolimus. Although this level is considered supraphysiological, our experimental design—specifically the preconditioning of MSCs prior to transplantation—prevents systemic exposure, thereby ensuring safety while maximizing efficacy [39]. The role of mTOR signaling in Akt activation in MSCs is controversial. During osteoblastic differentiation, activation of mTORC1 increases the expression of p16INK4a and cleaved caspase-3 (markers of senescence and apoptosis, respectively). These deleterious effects are alleviated by rapamycin, which not only inhibits mTORC1 but also indirectly enhances mTORC2/Akt signaling, thereby exerting anti-apoptotic effects via FOXO suppression [40, 41]. In contrast, under high glucose conditions, MSCs exhibit cellular senescence with increased expression of p-Akt (Ser 473), a downstream effector of mTORC2, and inhibiting this signal rescues the cells [33]. Therefore, the inhibition of mTORC2/Akt signaling is necessary to maintain the viability of MSCs under high glucose conditions.

In the present study, two in vivo experiments were performed using subconjunctival injection instead of the commonly used intravitreal injection method for drug delivery in DR. While intravitreal injection remains the clinical standard and achieves high intraocular concentrations, it carries risks such as retinal detachment, cataract formation, and, in some reports, epiretinal membrane formation or proliferative vitreoretinopathy due to cell aggregation [42–45]. Conversely, subconjunctival injection enables prolonged ocular retention compared with topical administration and effectively delivers drugs to the retina and vitreous body through trans-scleral diffusion [46, 47]. It is also less invasive than anterior chamber injections and provides a safer alternative to systemic administration, which often results in off-target exposure and systemic side effects [48]. Therefore, subconjunctival injection was selected as the administration route in the present study.

It is well established that retinal neuronal apoptosis and functional abnormalities occur even before clinically detectable DR [49]. In patients with long-term type 1 diabetes without DR, a significant reduction in b-wave amplitude has been reported, whereas the a-wave amplitude remained unchanged [50]. Another study demonstrated significant reductions in ERG amplitudes induced by high-frequency flicker stimuli (62.5 Hz) compared with standard flicker stimuli (31.25 Hz) in diabetic patients without DR or with mild non-proliferative DR [51]. Since the a-wave predominantly reflects photoreceptor activity, while the b-wave represents inner retinal function mediated by ON-bipolar and Müller cells, these findings suggest that structural recovery of photoreceptors does not necessarily translate into substantial functional improvement in early DR, whereas preservation of b-wave activity more closely parallels the maintenance of inner retinal integrity [52]. Supporting this interpretation, histopathological and mechanistic studies in diabetic models have revealed degeneration of photoreceptor outer segments and increased oxidative stress originating from the photoreceptor layer, highlighting its particular vulnerability during DR progression [53–55]. Accordingly, our therapeutic strategy appears especially effective in preventing or restoring inner retinal function, as further evidenced by histological findings showing restoration of total retinal and PRL thickness in the treated groups.

Although the direct effect of everolimus on glucose metabolism in high glucose–exposed MSCs remains unclear, mTOR signaling is known to regulate glucose metabolism across various cell types [56, 57]. In β-cells, hyperglycemia causes accumulation of glycolytic intermediates between PFK and GAPDH, which inhibits AMPK and activates mTORC1, thereby reducing pyruvate entry into the TCA cycle and lowering ATP production [56]. Similarly, in psoriasiform keratinocytes, high glucose and lipid exposure activated Akt/mTOR signaling, increasing glycolysis-related proteins such as membrane-GLUT1, HK2, and PFKFB3, while OXPHOS remained unchanged [57]. In the present study, everolimus treatment did not markedly alter glucose-metabolizing enzyme expression in UCB-MSCs under high glucose conditions. However, a decrease in the expression of PKLR, which encodes pyruvate kinase (PK), was observed [58]. PK expression is often enhanced by mTOR signaling, and in cancer cells, PK overexpression can promote ROS production by hyperactivating mitochondrial membrane potential [58, 59]. Conversely, in HeLa cells, PKM2 inhibited mitochondrial PDH through HIF1/PDK1 activation, leading to reduced TCA cycle activity and subsequent ROS generation [58]. Despite these findings, our results only detected modest changes in OXPHOS, represented by mitochondrial metabolism, and further studies are needed to confirm whether altered PK activity alone is sufficient to influence cellular metabolism. This leads us to focus on a more proximal mechanism: regulation of glucose uptake via GLUT3 trafficking rather than enzyme-level metabolic reprogramming.

Mechanistically, our data suggest that high glucose activates mTOR signaling to increase p-cofilin and stabilize actin, promoting GLUT3 membrane trafficking; everolimus reverses this effect. Both mTORC1 and mTORC2 regulate cytoskeletal remodeling, but through distinct mechanisms. mTORC2 interacts with focal adhesion proteins (FAK, paxillin, p130cas) together with Rho GTPase, PKCα, and Akt to control cell migration and actin polymerization [60]. In contrast, mTORC1-driven p-S6K1 signaling enhances chemotaxis and actin production in neutrophils and increases p-FAK expression in mechanically stimulated MSCs, independent of GSK-3β or PI3K/Akt [61, 62]. These findings suggest that actin remodeling is not restricted to a single mTOR complex, which may explain the ongoing debate regarding their relative contributions to GLUT trafficking [63, 64].

High glucose further activates the PKC/Rho pathway, leading to phosphorylation of MYPT, LIMK, and ERM [65]. In particular, p-LIMK inactivates cofilin, stabilizing actin filaments, a process linked to increased membrane GLUT1 expression in podocytes [65, 66]. Consistent with this, we observed elevated membrane GLUT3 levels along with increased p-cofilin under hyperglycemic conditions. Other contexts also highlight the importance of cofilin-mediated actin turnover in GLUT trafficking. In skeletal muscle, insulin stimulates Slingshot phosphatase via PAK1, resulting in cofilin dephosphorylation and rapid actin remodeling that drives GLUT4 translocation [67]. Conversely, in triple-negative breast cancer, activation of the PAK1/LIMK1/cofilin1 axis by sphingosine kinase 2 promotes cell migration rather than glucose uptake [68]. Although the diabetes-specific GLUT3 exocytosis signaling process remains unclear, in a model of diabetic nephropathy, lysine kinase, activated by PI3K/Akt signaling upregulation, binds the phosphoprotein-binding-14-3-3 adaptor molecule through TBC1D4 phosphorylation, which converts Rab8A to its GTP-bound form and translocates GLUT1-containing vesicles to the cell membrane [69, 70]. Taken together, these results suggest that the molecular mechanism of GLUT membrane trafficking mediated by actin turnover is highly cell-specific and varies in the diabetic milieu.

We demonstrated that everolimus regulates actin-mediated GLUT3 membrane trafficking by inhibiting mTOR signaling, thereby blocking glucose influx, mitigating mtROS overproduction, and reversing high glucose-induced cell death and dysfunction in UCB-MSCs. To our knowledge, this is the first study describing the detailed mechanism of GLUT3-driven mtROS generation in UCB-MSCs under high glucose conditions. We further demonstrated that cofilin activation by everolimus plays an important role in retrieving GLUT3 from the membrane. Therefore, we propose a promising new MSC-based therapeutic strategy involving the subconjunctival injection of everolimus-pretreated UCB-MSCs to restore retinal tissue structure and function in DR patients.

## Supplementary information

Supplementary materials

Original Western blot

## Data Availability

Data are included within the article. The original data of Western blots are all provided. The microarray data generated in this study have been deposited in the NCBI Gene Expression Omnibus (GEO) under accession number GSE308339.
