# Supplementary material for: Everolimus suppresses glucose transporter 3 membrane trafficking to improve therapeutic efficacy of umbilical cord blood-derived mesenchymal stem cell transplantation in diabetic retinopathy
Source: Cell Death Dis. 2026 Mar 28;17(1):426. doi: 10.1038/s41419-026-08673-6 (PMC13153175; doi:10.1038/s41419-026-08673-6)
Supplement: Supplementary file 1 — Supplementary materials [file 41419_2026_8673_MOESM1_ESM.docx]

**Title: Everolimus Suppresses Glucose Transporter 3 Membrane Trafficking to Improve Therapeutic Efficacy of Umbilical Cord Blood-derived Mesenchymal Stem Cell Transplantation in Diabetic Retinopathy

Hyo Youn Jo** ^1,2,*^**, Ji Seung Jung** ^3,*^**, Hang Hyo Jo** ^1,2,*^**, Dae Hyun Kim** ^1,2^**, Yeon Ju Oh** ^1,2^**, Jiyi Hwang** ^3^**, Kyung-Mee Park** ^3,†^**, and Hyun Jik Lee** ^1,2,†^ **Affiliations:**^1^ Laboratory of Veterinary Physiology, College of Veterinary Medicine and Veterinary Medicine Center, Chungbuk National University, Cheongju 28644, Republic of Korea
^2^ Institute for Stem Cell & Regenerative Medicine (ISCRM), Chungbuk National University, Cheongju 28644, Republic of Korea.
^3^ Laboratory of Veterinary Surgery and Ophthalmology, College of Veterinary Medicine and Veterinary Medicine Center, Chungbuk National University, Cheongju 28644, Republic of Korea

**E-mail addresses:**
Hyo Youn Jo: hyoyun05@chungbuk.ac.kr
Ji Seung Jung: wjdwltmd00@gmail.com
Hang Hyo Jo: hyo1126@chungbuk.ac.kr
Dae Hyun Kim: woobboy1990@hotmail.com
Yeon Ju Oh: oyj723@naver.com
Jiyi Hwang: wldml1013@gmail.com
Kyung-Mee Park: parkkm@cbnu.ac.kr
Hyun Jik Lee: leehyunjik@chungbuk.ac.kr

^*^ These authors contributed equally to this work.

^†^Correspondence: Kung-Mee Park and Hyun Jik Lee

^†^Kyung-Mee Park, D.V.M., Ph.D.
College of Veterinary Medicine and Veterinary Medicine Center, Chungbuk National University, Cheongju 28644, Republic of Korea
Tel: +82-043-261-2985 / E-mail: parkkm@cbnu.ac.kr

^†^Hyun Jik Lee, D.V.M., Ph.D.
College of Veterinary Medicine and Veterinary Medicine Center, Chungbuk National University, Cheongju 28644, Republic of Korea
Tel: +82-043-261-2597 / E-mail: leehyunjik@chungbuk.ac.kr

Running Title: Therapeutic Effect of Everolimus-pretreated UCB-MSCs for Diabetic Retinopathy

**Supplementary Table S1. Sequences of primers used for real-time qPCR**

| Gene | Forward (5’ – 3’) | Reverse (5’ – 3’) |
| --- | --- | --- |
| *GLUT1* | CTTCACTGTCGTGTCGCTGT | CCAGGACCCACTTCAAAGAA |
| *GLUT3* | ACCGGCTTCCTCATTACCTT | AGGCTCGATGCTGTTCATCT |
| *SOD1* | GTAGTCTCCTGCAGCGTCTG | ATGCAGGCCTTCAGTCAGTC |
| *SOD2* | GGCCTACGTGAACAACCTGA | GAAACCAAGCCAACCCCAAC |
| *CAT* | AGTGATCGGGGGATTCCAGA | AAGTCTCGCCGCATCTTCAA |
| *ACTB* | CCACCATGTACCCTGGCATT | CGGACTCGTCATACTCCTGC |

**Supplementary Table S2. Sequences of siRNAs used for gene silencing**

| Gene | Forward (5’ – 3’) | Reverse (5’ – 3’) |
| --- | --- | --- |
| *GLUT1* | GUAUACUACUGCUUCAUCU | AGAUGAAGCAGUAGUAUAC |
|  | GUGAUCGAGGAGUUCUACA | UGUAGAACUCCUCGAUCAC |
|  | CUCUUCCUACCCAACCACU | AGUGGUUGGGUAGGAAGAG |
| *GLUT3* | CCGCUGCUACUGGGUUUUA | UAAAACCCAGUAGCAGCGG |
|  | CUGUGUAAAGUAGCUAAGU | ACUUAGCUACUUUACACAG |
|  | GACUUUGGAUCCUUCCUGA | UCAGGAAGGAUCCAAAGUC |

**
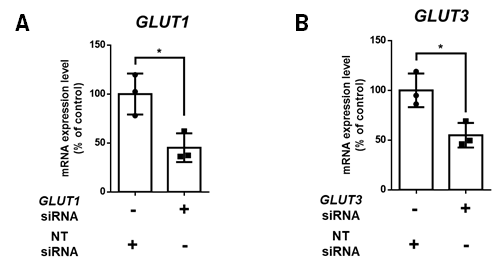
**

**Supplementary Fig. S1 Silencing efficacy of *GLUT1* and *GLUT3* siRNAs**. **(A)** UCB-MSCs were transfected with *GLUT1* siRNA or NT siRNA for 24 h, followed by incubation in serum-free medium for 72 h. *GLUT1* mRNA expression was quantified by real-time qPCR (*n* = 3). **(B)** UCB-MSCs were transfected with *GLUT3* siRNA or NT siRNA for 24 h, followed by incubation in serum-free medium for 72 h. *GLUT3* mRNA expression was quantified by real-time qPCR (*n* = 3). All quantitative data are presented as the mean ± standard deviation from independent experiments. **p* < 0.05.

**
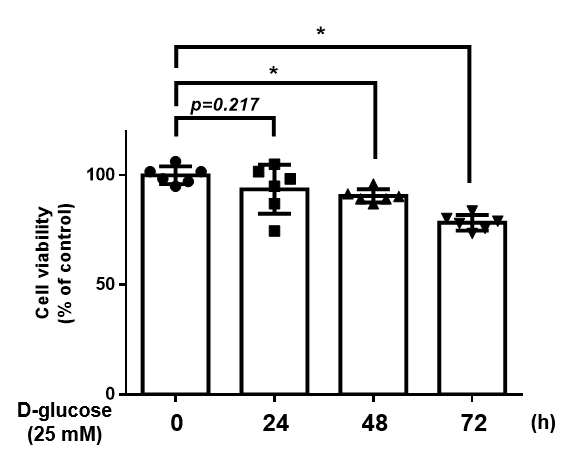
**

**Supplementary Fig. S2 High glucose reduces cell viability.** UCB-MSCs were treated with D-glucose (25 mM) at various times (0–72 h). Cell viability was measured using a trypan blue exclusion assay (*n* = 6). Quantitative data is presented as the mean ± standard deviation from independent experiments. **p* < 0.05.


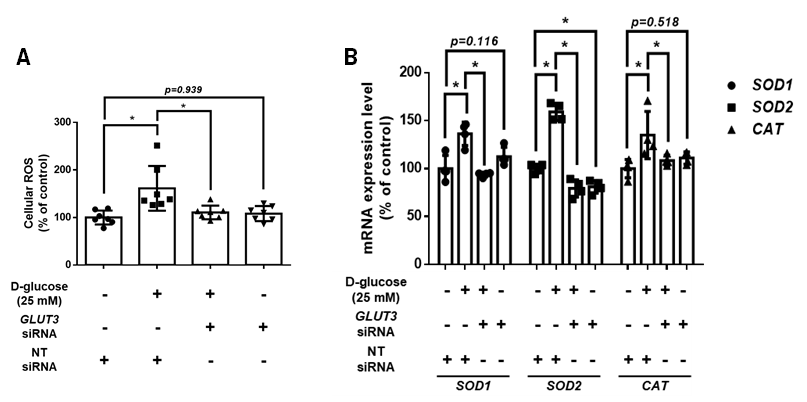


**Supplementary Fig. S3 Effects of GLUT3 silencing on cellular ROS generation and antioxidant enzyme expression. (A and B)** UCB-MSCs were transfected with *GLUT3* siRNA or NT siRNA for 24 h, followed by exposure to 25 mM of D-glucose for 72 h*.* **(A)** Cellular ROS was assessed by DCF-DA staining (*n* = 7). **(B)** The expression of *SOD1, SOD2* and *CAT* mRNA was measured by real-time quantitative PCR (*n =* 4). All quantitative data are presented as the mean ± standard deviation from independent experiments. **p* < 0.05.

**
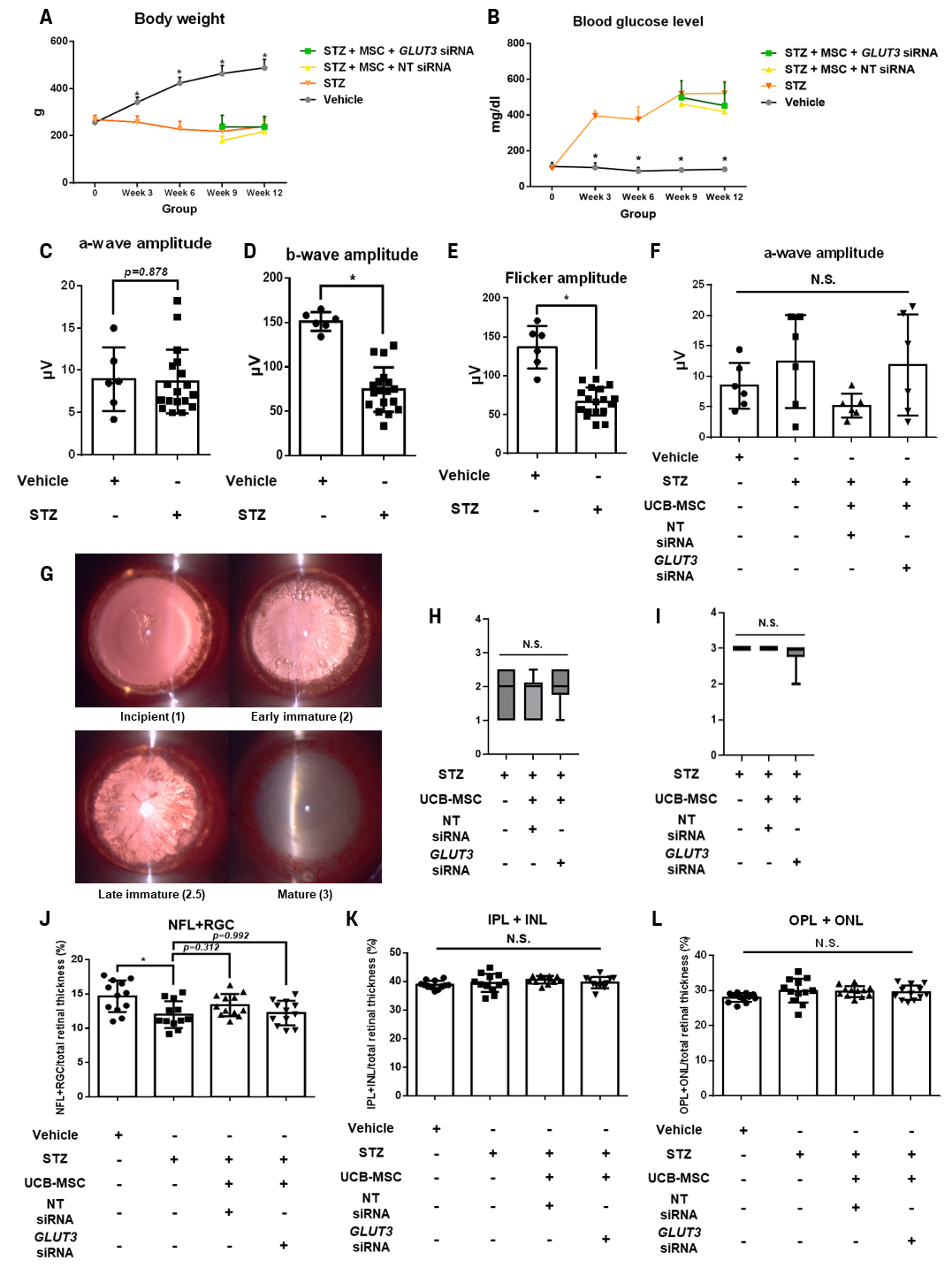
Supplementary Fig. S4 Effects of *GLUT3* knockdown on UCB-MSCs on diabetic retinal function and cataract formation. (A and B)** Body weight and blood glucose were measured to determine the systemic effects of the treatment. The measurements were taken at 0, 3, 6, 9, and 12 weeks post-STZ injection (*n* = 3–9). **(C–E)** Electroretinography (ERG) was performed at 8 weeks post-STZ injection to confirm retinal dysfunction caused by diabetic mellitus. It was measured in both eyes of all rats in each group. **(C)** a-wave amplitudes were compared between the groups (*n* = 6–18). **(D)** b-wave amplitudes were compared between the groups (*n* = 6–18). **(E)** Flicker amplitudes were compared between the groups (*n* = 6–18). **(F)** ERG was performed to assess treatment efficacy at 12 weeks post-STZ injection, and a-wave amplitudes were compared between the groups (*n* = 6). **(G–I)** Representative slit-lamp photographs illustrating cataract stages from incipient to mature (scores 1 to 3). **(H and I)** Cataract diagnosis following STZ injection was performed at two ERG recording time points. All rats in each group were evaluated for both eyes. **(H)** Cataract staging was performed among the groups after 8 weeks of STZ induction (*n* = 6). **(I)** Cataract staging was performed among the groups after 12 weeks of STZ induction (*n* = 6). **(J–L)** Retinal morphology was examined microscopically at 2 locations in both eyes of 3 rats in each group. **(J)** The nerve fiber layer and retinal ganglion cell layer were measured for each group (*n* = 12). **(K)** The inner plexiform layer and inner nuclear layer were measured for each experimental group (*n* = 12). **(L)** The outer plexiform layer and outer nuclear layer were measured for each experimental group (*n* = 12). All quantitative data are presented as the mean ± standard deviation or box-and-whisker plots with median and interquartile range. N.S., no significance. **p* < 0.05.


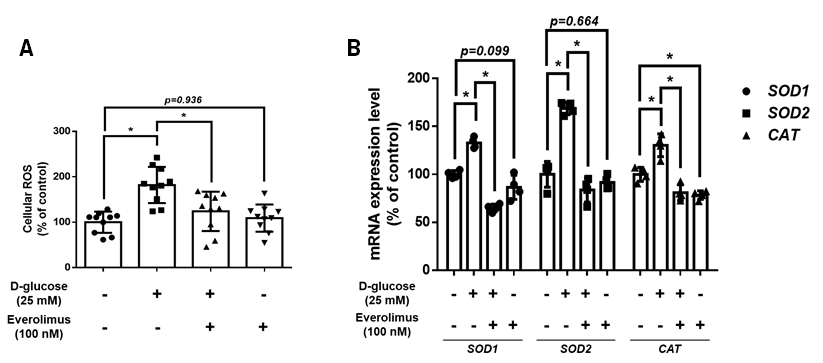


**Supplementary Fig. S5 Effects of everolimus on cellular ROS generation and antioxidant enzyme expression. (A and B)** UCB-MSCs were pretreated with everolimus (100 nM) for 30 min followed by D-glucose (25 mM) treatment for 72 h. **(A)** Cellular ROS was assessed by DCF-DA staining (*n* = 10). **(B)** The expression of *SOD1, SOD2* and *CAT* mRNA was measured by real-time quantitative PCR (*n =* 4). All quantitative data are presented as the mean ± standard deviation from independent experiments. **p* < 0.05.


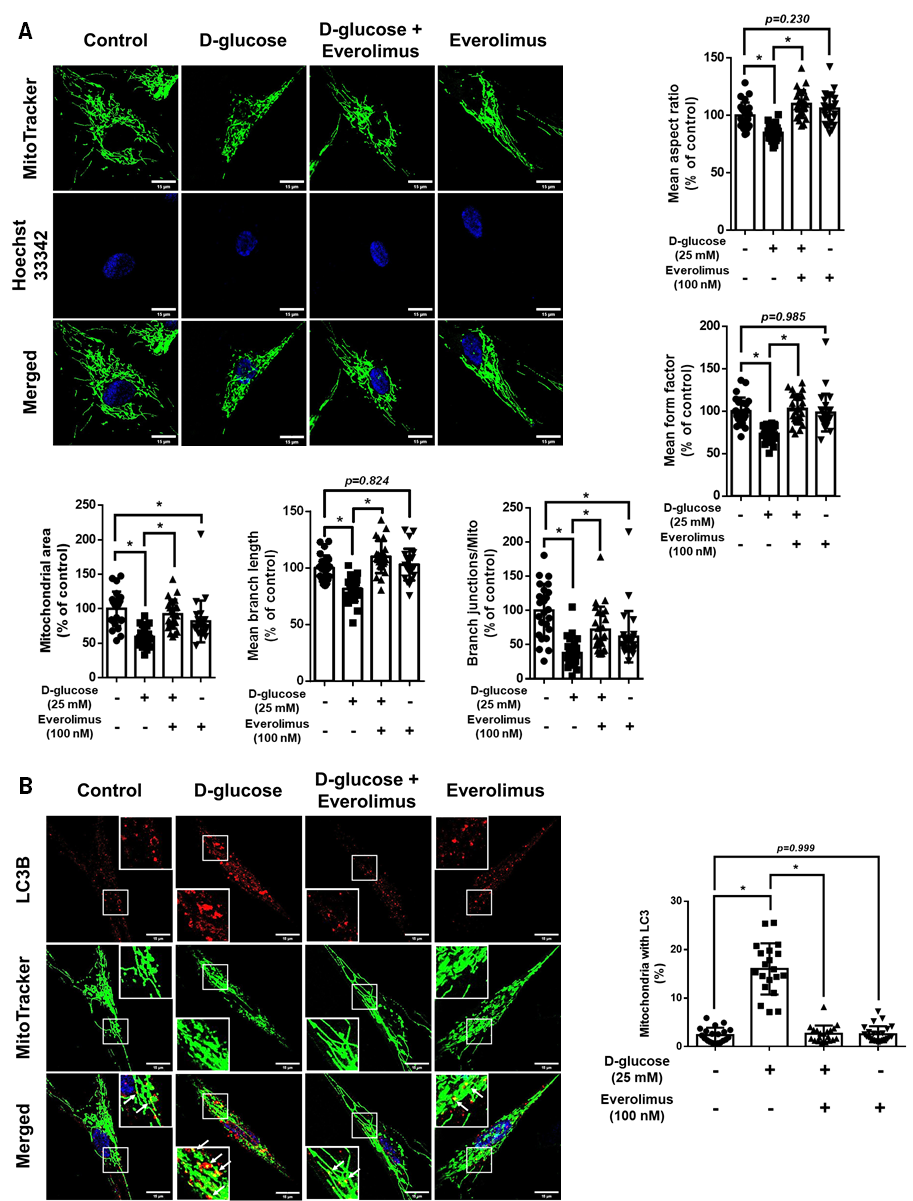


**Supplementary Fig. S6 Effects of everolimus on mitochondrial dynamics and mitophagy. (A and B)** UCB-MSCs were pretreated with everolimus (100 nM) for 30 min followed by D-glucose (25 mM) treatment for 72 h. **(A)** Mitochondrial fusion/fission was visualized by immunocytochemistry. UCB-MSCs were stained with a MitoTracker (green), and Hoechst 33342 (blue) (*n* = 25). Magnification × 1,500. Scale bars are 15 μm. **(B)** Mitophagy was visualized by immunocytochemistry. UCB-MSCs were stained with a LC3B-specific antibody (red), MitoTracker (green), and Hoechst 33342 (blue) (*n* = 20). Magnification × 1,500. Scale bars are 15 μm. All quantitative data are presented as the mean ± standard deviation from independent experiments. **p* < 0.05.


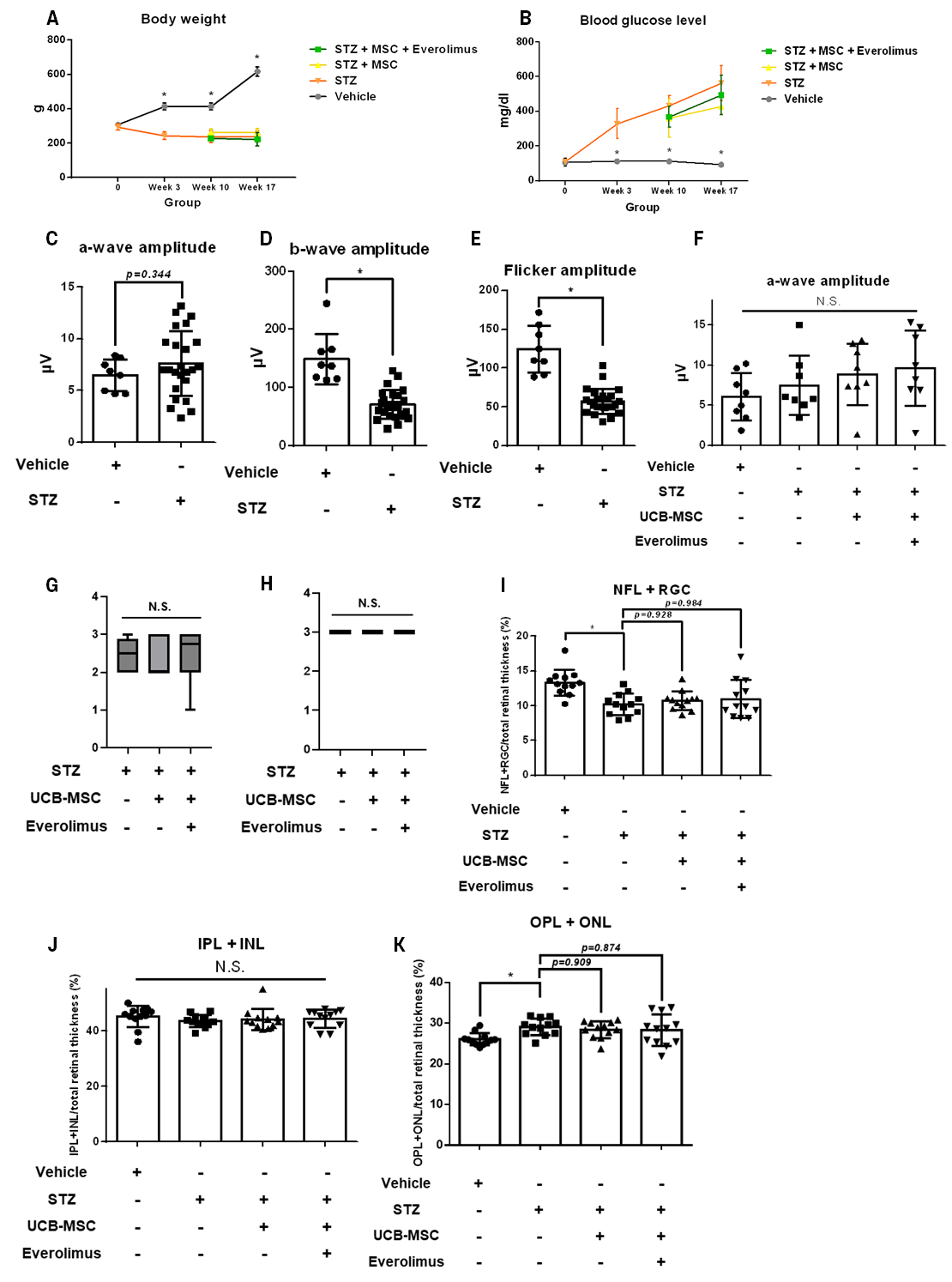


**Supplementary Fig. S7 Effects of everolimus-pretreated UCB-MSCs on retinal function and cataract formation in DR rats.** **(A and B)** Body weight and blood glucose were measured to determine the systemic effects of the experiment. These measurements were taken at 0, 3, 10, and 17 weeks post-STZ injection (*n* = 4–12). **(C–E)** Electroretinography (ERG) was performed at 10 weeks post-STZ injection to confirm retinal dysfunction caused by diabetic mellitus. It was measured in both eyes of all rats in each group. **(C)** a-wave amplitudes were compared between groups (*n* = 8–24). **(D)** b-wave amplitudes were compared between groups (*n* = 8–24). **(E)** Flicker amplitudes were compared between groups (*n* = 8–24). **(F)** ERG was performed to assess treatment efficacy at 14 weeks following STZ injection. a-wave amplitudes were compared between groups (*n* = 8). **(G and H)** Cataract diagnosis following STZ injection was performed at two ERG recording time points. All rats in each group were evaluated for both eyes. **(G)** Cataract staging was performed among the groups after 10 weeks of STZ induction (*n* = 8). **(H)** Cataract staging was performed among the groups after 14 weeks of STZ induction (*n* = 8). **(I–K)** Retinal morphology was examined microscopically at 2 locations in both eyes of 3 rats in each group. **(I)** The nerve fiber layer and retinal ganglion cell layer were measured for each group (*n* = 12). **(J)** The inner plexiform layer and inner nuclear layer were measured for each group (*n* = 12). **(K)** The outer plexiform and outer nuclear layers were measured for each group (*n* = 12). All quantitative data are presented as the mean ± standard deviation or box-and-whisker plots with median and interquartile range. N.S., no significance. **p* < 0.05.


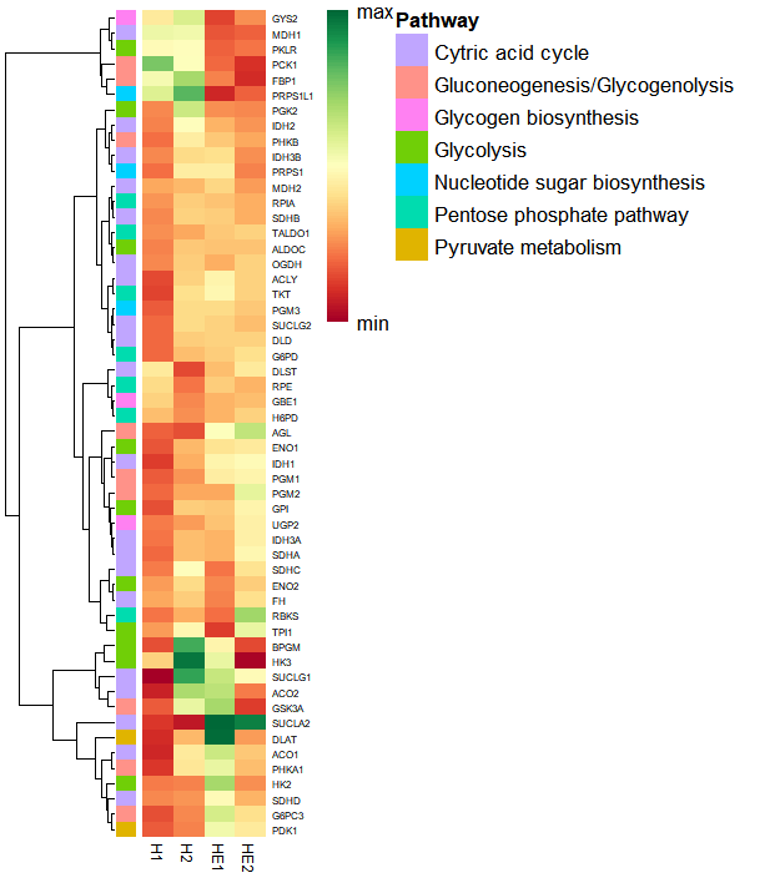


**Supplementary Fig. S8 Effect of everolimus on glucose metabolism-related enzymes in UCB-MSCs.** UCB-MSCs were pretreated with everolimus (100 nM) for 30 min followed by D-glucose (25 mM) treatment for 72 h. Total RNA was extracted to analyze the expression of glucose metabolism-related gene mRNAs using a microarray. Heatmap with hierarchical clustering shown in the left panel was acquired using the pheatmap package on R studio. Up- and down-regulated genes with a fold-change >1.5 and a *p-value* <0.05 were selected (*n* = 2).


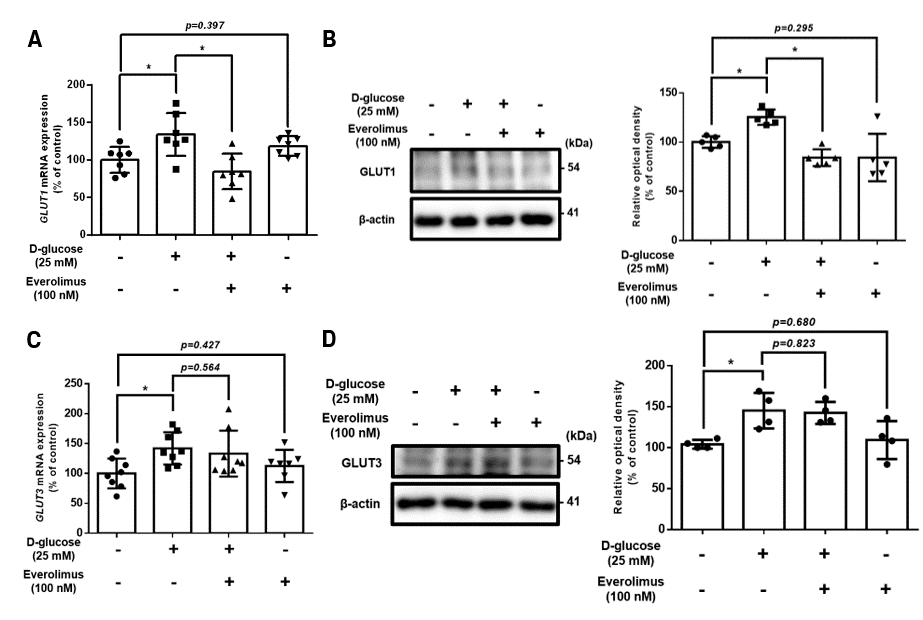


**Supplementary Fig. S9 Role of everolimus in GLUT1 and GLUT3 expression. (A–D)** UCB-MSCs were pretreated with everolimus (100 nM) for 30 min followed by D-glucose (25 mM) treatment for 72 h. **(A)** The expression of *GLUT1* mRNA was analyzed using real-time quantitative PCR (*n* = 7). **(B)** Protein expression levels of GLUT1 were determined by western blot analysis (*n* = 5). **(C)** The expression of *GLUT3* mRNA was analyzed using real-time quantitative PCR (*n* = 8). **(D)** GLUT3 protein expression was determined by western blot analysis (*n* = 4). **p* < 0.05.


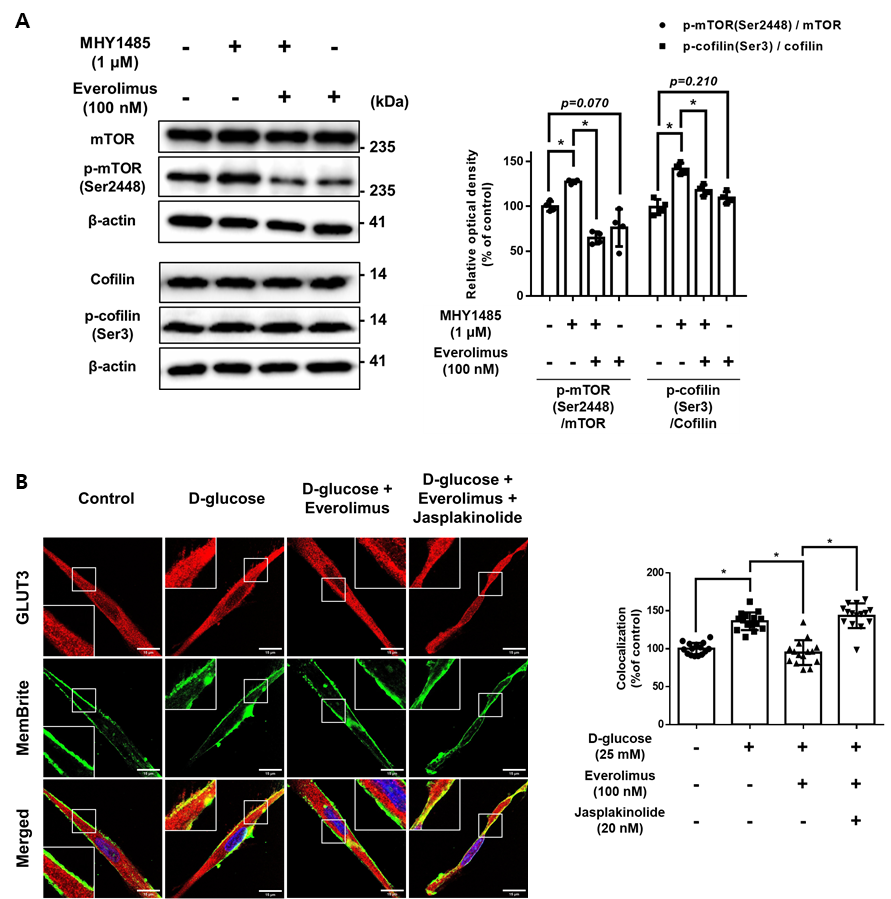


**Supplementary Fig. S10 Effects of mTOR–cofilin signaling on actin remodeling and GLUT3 membrane localization. (A)** UCB-MSCs were pretreated with everolimus (100 nM) for 30 min followed by MHY1485 (1 μM) treatment for 72 h. Protein expression levels of mTOR, p-mTOR (Ser 2448), cofilin, and p-cofilin (Ser 3) were determined by western blot analysis (*n* = 4). **(B)** UCB-MSCs were pretreated with jasplakinolide (20 nM) for 30 min followed by everolimus (100 nM) with D-glucose (25 mM) treatment for 72 h. Membrane trafficking of GLUT3 was visualized by immunocytochemistry. UCB-MSCs were stained with a GLUT3-specific antibody (red), MemBrite (green), and DAPI (blue) (*n* = 15). Magnification × 1,500. Scale bars are 15 μm. All quantitative data are presented as the mean ± standard deviation from independent experiments. **p* < 0.05.
