# Supplementary figures and images for: Everolimus suppresses glucose transporter 3 membrane trafficking to improve therapeutic efficacy of umbilical cord blood-derived mesenchymal stem cell transplantation in diabetic retinopathy
Source: Cell Death Dis. 2026 Mar 28;17(1):426. doi: 10.1038/s41419-026-08673-6 (PMC13153175; doi:10.1038/s41419-026-08673-6)

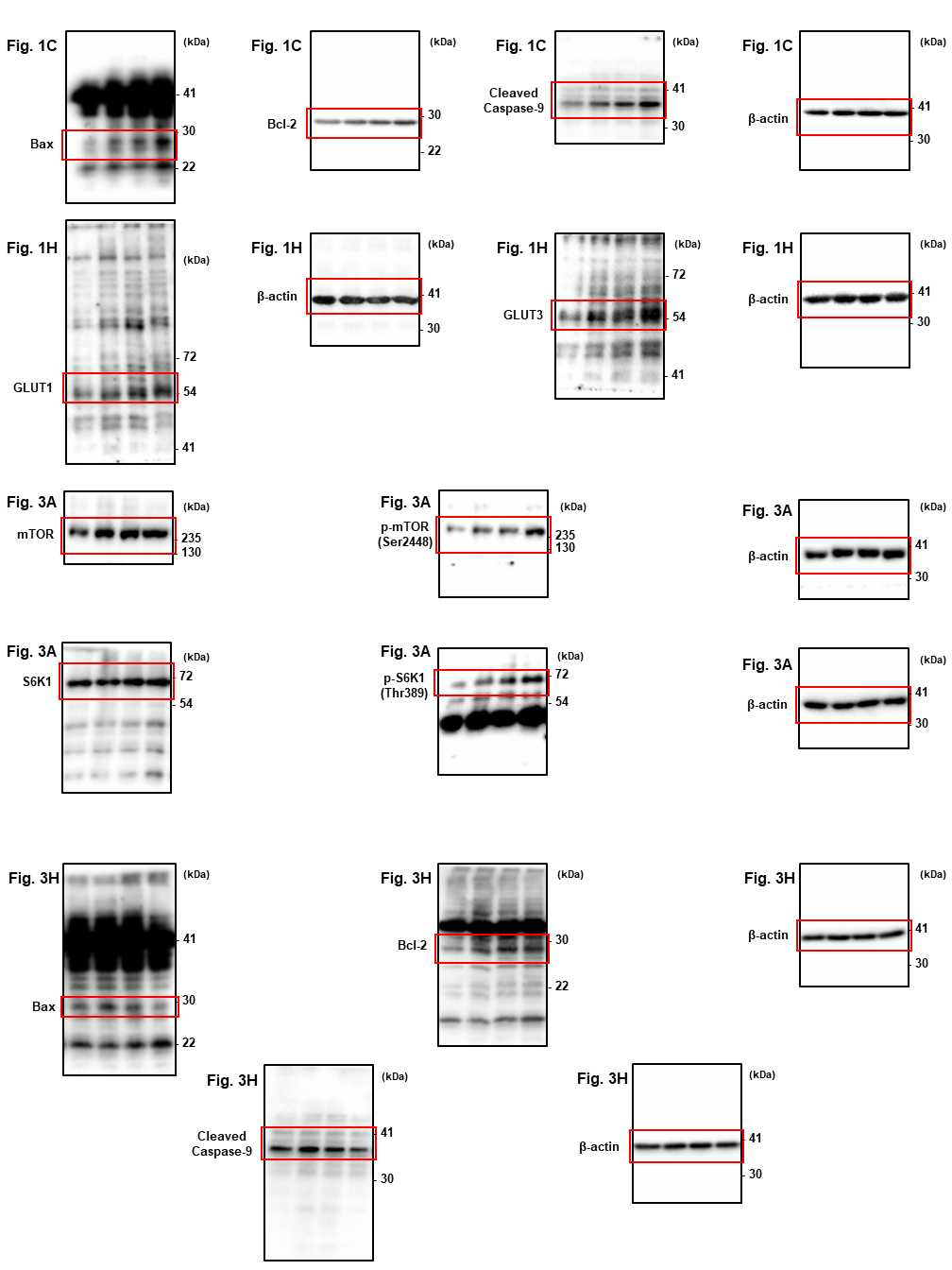

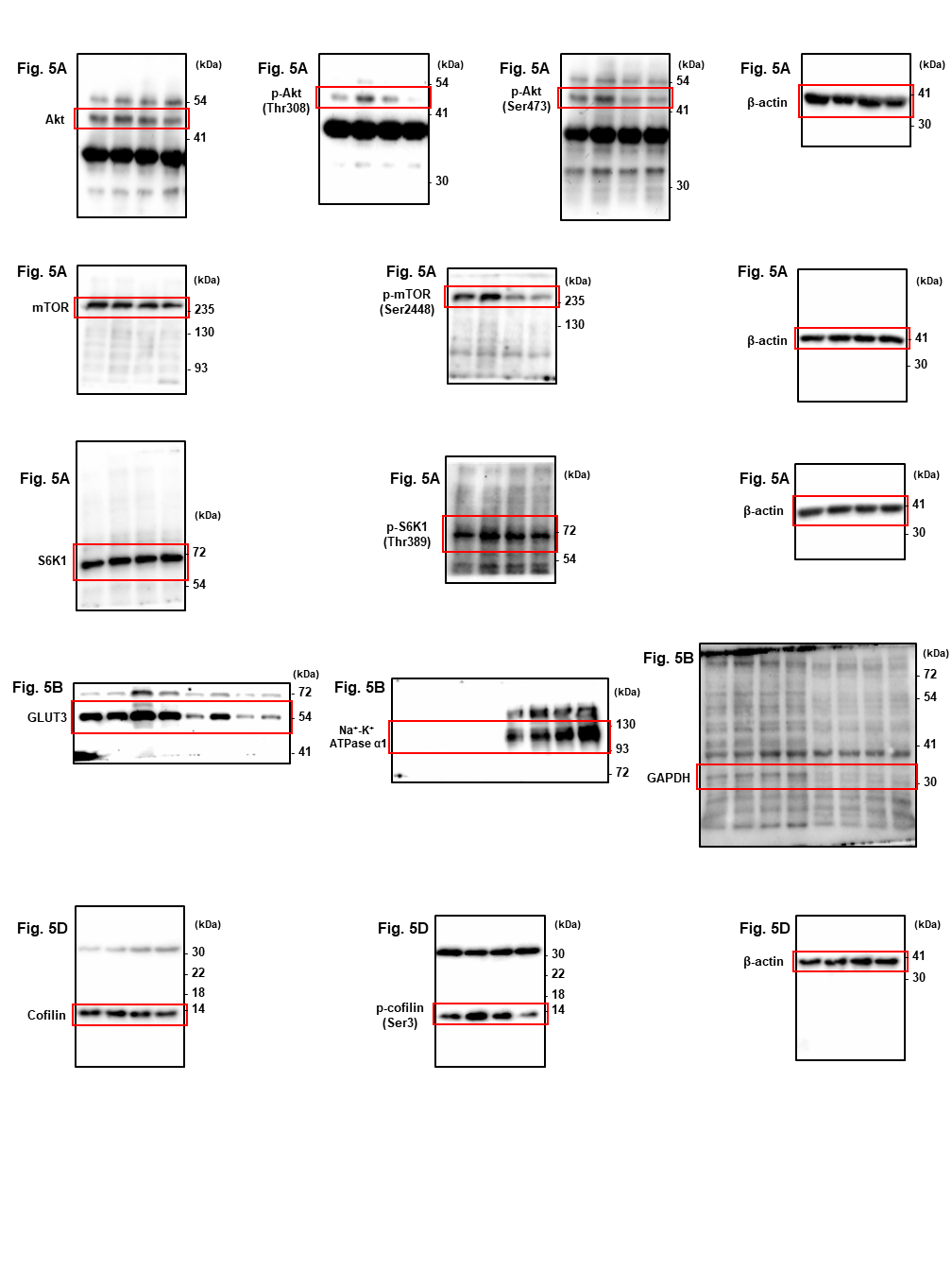

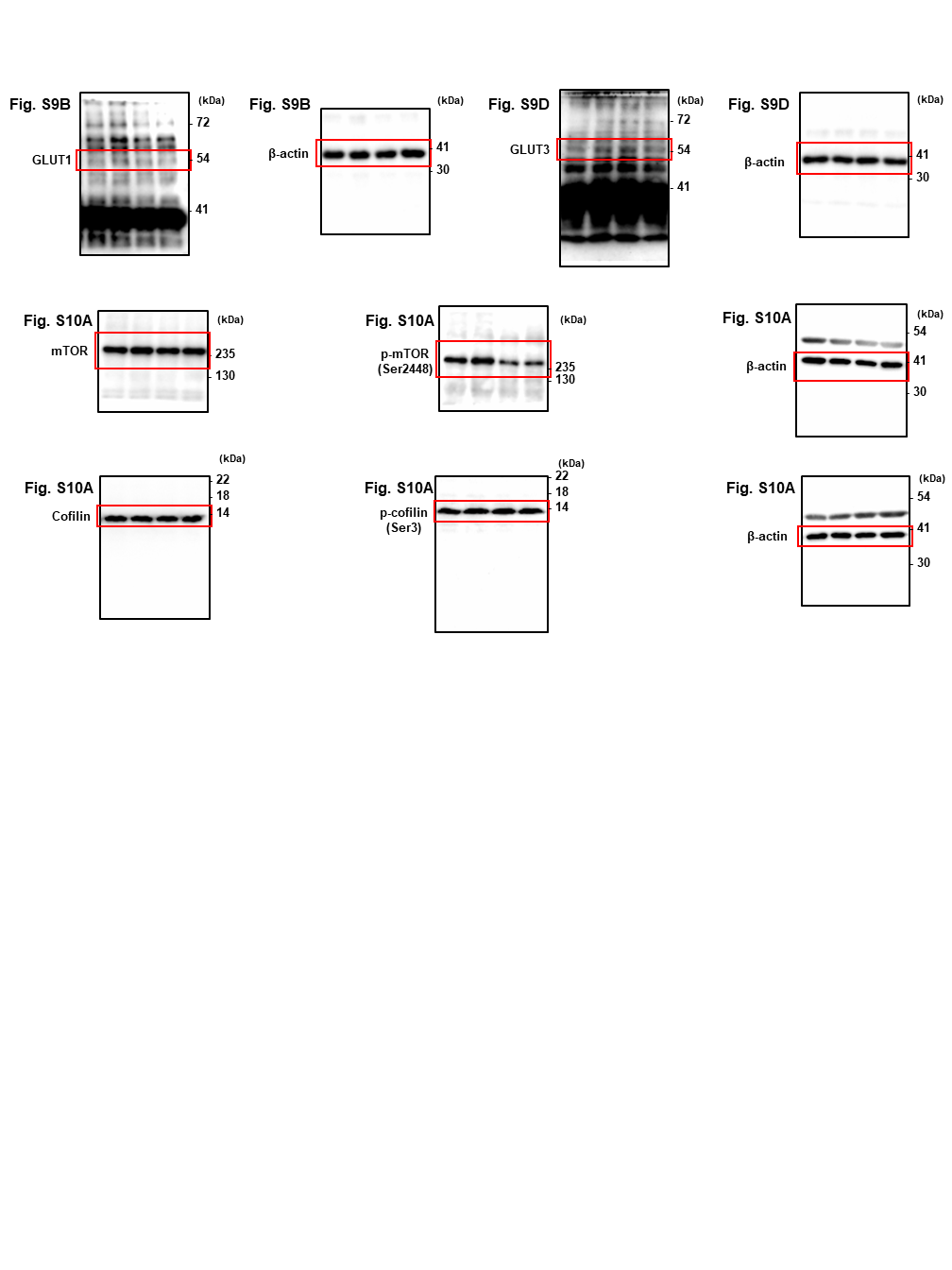

Supplement: Supplementary file 2 — Original Western blot [file 41419_2026_8673_MOESM2_ESM.docx]
